# Transient DREADD Manipulation of the Dorsal Dentate Gyrus in Rats Impairs Initial Learning of Place‐Outcome Associations

**DOI:** 10.1002/hipo.70014

**Published:** 2025-05-06

**Authors:** J. Lim, A. Souiki, P. Ahmad, C. A. Oomen, G. J. Huis in ’t Veld, C. S. Lansink, C. M. A. Pennartz, U. Olcese

**Affiliations:** ^1^ Cognitive and Systems Neuroscience Group Swammerdam Institute for Life Sciences, University of Amsterdam Amsterdam the Netherlands

**Keywords:** dentate gyrus, DREADDs, episodic memory, hippocampus, place‐reward associations, rats

## Abstract

The dentate gyrus subfield of the hippocampus is thought to be critically involved in the disambiguation of similar episodic experiences and places in a context‐dependent manner. However, most empirical evidence has come from lesion and gene knock‐out studies in rodents, in which the dentate gyrus is permanently perturbed and compensation of affected functions via other areas within the memory circuit could take place. The acute and causal role of the dentate gyrus herein remains therefore elusive. The present study aimed to investigate the acute role of the dorsal dentate gyrus in disambiguation learning using reversible inhibitory DREADDs. Rats were trained on a location discrimination task and learned to discriminate between a rewarded and unrewarded location with either small (similar condition) or large (dissimilar condition) separation. Reward contingencies switched after applying a reversal rule, allowing us to track the temporal engagement of the dentate gyrus during the task. Bilateral DREADD modulation of the dentate gyrus impaired the initial acquisition learning of place‐reward associations, but performance rapidly recovered to baseline levels within the same session. Modeling of the behavioral patterns revealed that reward sensitivity and alternation behavior were temporally associated with the DG‐dependent impairment during acquisition learning. Our study thus provides novel evidence that the dorsal dentate gyrus is acutely engaged during the initial acquisition learning of place‐reward associations.

## 1 Introduction

Remembering places associated with salient events and keeping these memories distinct when encountering similar spatial contexts are important cognitive functions to guide spatial navigation and decision making. The hippocampus is critically involved in memory (Eichenbaum et al. 1996), spatial navigation (O'Keefe and Dostrovsky 1971; O'Keefe 1976; O'Keefe and Nadel 1978; McNaughton et al. 1996), and spatial memory (Eichenbaum et al. 1990; McDonald and White 1995). Specifically, the hippocampus encodes individual places, trajectories and environments and their temporally associated sensory, motivational and more abstract events (e.g., place‐reward associations) in a context‐dependent manner (Leutgeb et al. 2007; Lansink et al. 2009; reviewed in Lisman et al. 2017; Latuske et al. 2018; van Dijk and Fenton 2018). The mapping of highly similar contexts onto distinct neural representations by the hippocampus is crucial to successfully discriminate between contexts and reflects a more general process referred to as pattern separation. The ability to disambiguate similar experiences is thought to be degraded in the early stages of Alzheimer's disease (Ally et al. 2013; Wesnes et al. 2014; Zhu et al. 2017; Leal and Yassa 2018; Lee et al. 2020; Parizkova et al. 2020; Laczó et al. 2021), emphasizing the need to understand the neural basis of pattern separation.

The dentate gyrus subfield (DG) of the hippocampus is thought to be critically involved in pattern separation of similar contexts by orthogonalizing input activity patterns into separate, distinct neural representations (Marr 1971; Treves and Rolls 1994; Hunsaker and Kesner 2013; Knierim and Neunuebel 2016). Empirical evidence for this disambiguation function attributed to the DG has mostly come from behavioral lesion studies in non‐human primates (Hampton and Murray 2004; Lavenex et al. 2006) and rodents (Gilbert et al. 2001; Hunsaker and Kesner 2008; Morris et al. 2012; Lee and Solivan 2010), as well as gene knock‐out studies in rodents (McHugh et al. 2007; Kannangara et al. 2015; Yun et al. 2023). In these studies, dysfunction of the dorsal DG (dDG) was typically associated with an impaired ability to discriminate between adjacent positions (Gilbert et al. 2001; Hunsaker and Kesner 2008; McHugh et al. 2007; Morris et al. 2012; Kannangara et al. 2015; Oomen et al. 2015; Yun et al. 2023) and object locations (Lee and Solivan 2010), with animals making more mistakes in identifying the location associated with reward. Although the general conclusion from these studies is that dDG dysfunction impairs the ability to spatially separate the stimulus locations from one another, it remains difficult to interpret the impact of lesions on behavioral discrimination, given that lesions are often crude and permanent and may result in compensation of affected functions via other areas.

**FIGURE 1:**
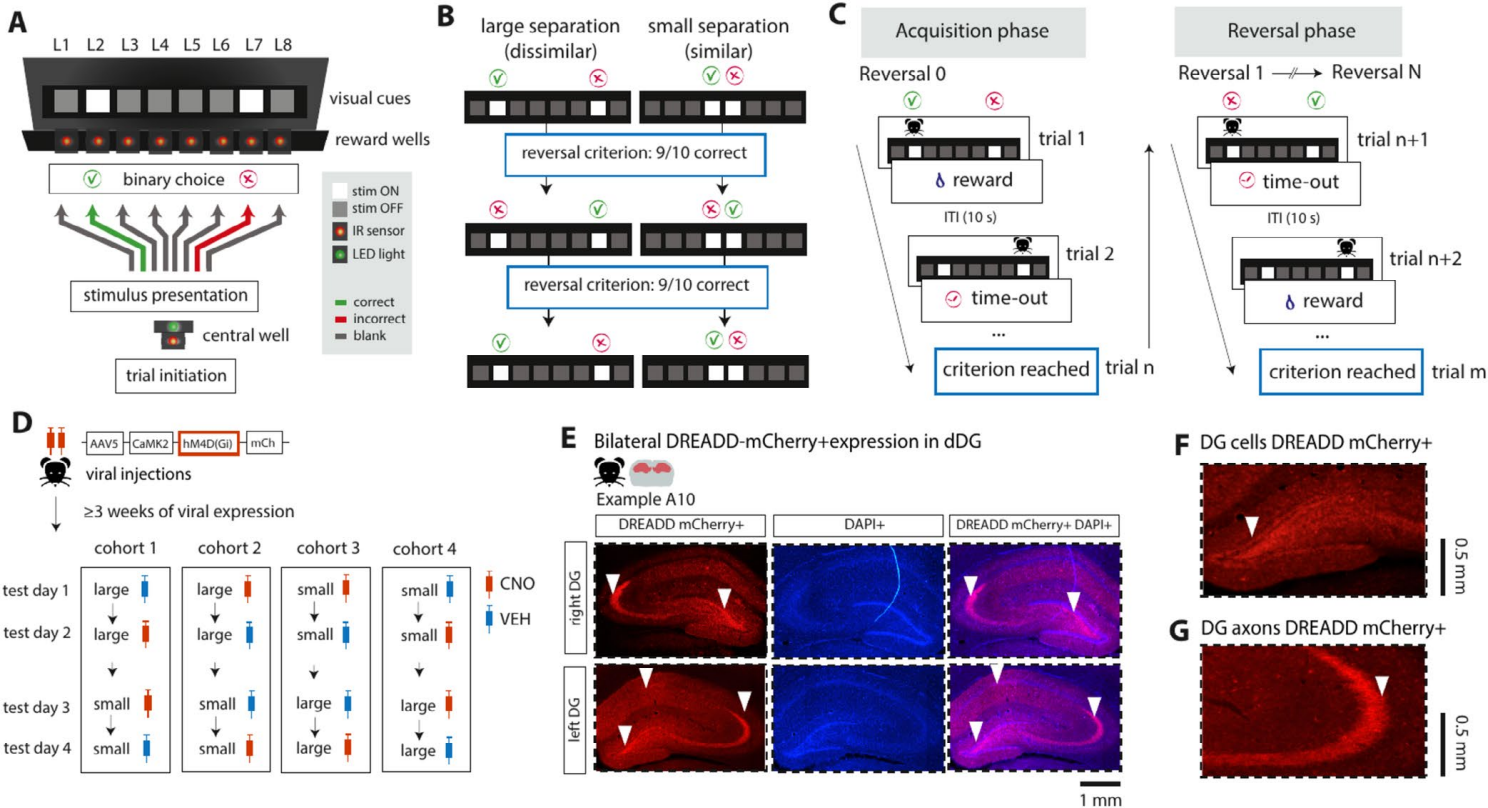
Task design of location discrimination task with chemogenetic silencing of dorsal dentate gyrus. (A) Schematic overview of operant box with the CS+ and CS− visual cues (white squares) presented on a black monitor screen. After trial initiation at the central well, animals made a binary choice (left or right) with a nose poke in the corresponding cued nose poke well. (B) Cued stimuli were presented with a large separation or small separation. After 9/10 consecutive correct choices, the reward contingencies switched. (C) Schematic illustration of the learning phases of the task. At acquisition, animals identified the rewarded location by trialanderror with positive (sucrose reward) and negative (time‐out) feedback. In the reversal phase, animals re‐learnt to identify the reversed target locations by adapting their choice behavior. (D) Chemogenetic targeting of the DG. Animals were bilaterally injected in the dDG with DREADD‐mCherry (*N* = 20). At testing, animals were injected with either CNO or saline (VEH) 30 min. before testing in a balanced within‐subject design. (E) Histological verification of an example animal (A10) with bilateral DREADD‐mCherry+ expression in dDG (left panel), DAPI expression (middle panel) and overlaid (right panel). White triangles indicate subregions of the hippocampus expressing DREADDs. (F) Close up of DREADD‐mCherry+ expression in the hilus and granule cell layer of the dDG, and (G) DG‐projecting axons in CA3.

To better understand the acute and causal engagement of the DG in spatial disambiguation learning in tasks designed to engage behavioral discrimination with more spatial and temporal control, we transiently disrupted the dDG with an inhibitory Designer Receptors Exclusively Activated by Designer Drugs compound (DREADDs; Roth 2016) in rats performing a location discrimination task (Figure 1A; Oomen et al. 2013; Oomen et al. 2015). Specifically, we targeted the excitatory cell population in the dDG, consisting of granule cells and mossy cells, with the former densely projecting to area CA3. The behavioral task probed spatial learning in rats discriminating between a rewarded and an unrewarded location. To track the temporal engagement of the dDG and avoid spatial biases to one side of the box, reward contingencies reversed after every 9 out of 10 consecutive correct responses (Figure 1B,C; Oomen et al. 2013). We found a significant impairment of discrimination performance when the DG was disrupted, but only during the acquisition phase of the task; performance recovered to baseline levels within a test session. To understand which aspects of learning were affected, we applied a behavioral model suitable to the task at hand and able to probe learning parameters of behavior (Sutton and Barto 2018; Metha et al. 2020). Specifically, we quantified the trial‐to‐trial reward‐driven learning with a reinforcement learning (RL) model and the tendency to persevere in choice behavior with a perseverance RL (PRL) model. These behavioral models revealed that the DG‐dependent acquisition deficit was temporally associated with decreased sensitivity to reward and increased alternation behavior. Our study thus provides novel evidence that the dDG is acutely but transiently engaged during spatial discrimination learning.

## 2 Methods

### 2.1 Animals

Twenty male Lister‐Hooded rats (Envigo, the Netherlands), aged 7–8 weeks at arrival, were housed in pairs in standard polycarbonate cages on a reversed 12‐h day‐light cycle. Animals were habituated to their home cages with *ad libitum* water and food and were handled daily by the researchers for a period of 1 week. To motivate animals to perform the behavioral task, animals were food restricted to maximally 85% of the standard growth curve as obtained under *ad libitum* feeding conditions and had *ad libitum* access to water. All experimental procedures were performed in accordance with the European Directive 2010/63/EU, guidelines of the Federation of European Laboratory Animal Science Associations, the NIH Guide for the Care and Use of Laboratory Animals, and were approved by the national and institutional ethics committees.

### 2.2 Behavioral Task With DREADD Manipulation

To probe spatial pattern separation, animals were trained and tested on a location discrimination (LD) task (Figure 1A). The LD task was adapted from the touchscreen LD operant platform task for testing hippocampus‐dependent spatial memory in both rats (McTighe et al. 2009; Svensson et al. 2016) and mice (Clelland et al. 2009; Creer et al. 2010; Coba et al. 2012). In our LD task, the reward wells were positioned underneath each of the 8 stimulus loci presented on a screen, instead of a sole reward well located at the side opposite to the screen wall (Figure 1A; as in McTighe et al. 2009). This design reduced training times of rats (C.A.O., unpublished observations), likely because the spatially aligned stimulus and reward locations allowed for a more rapid formation of spatial stimulus–outcome associations. Moreover, rats performed self‐initiated trials, facing the screen using a central nose poke well to ensure that animals were clearly exposed to the stimuli at trial onset.

Animals were trained in an automated operant box (52 cm width × 40 cm height × 50 cm depth) with three black aluminium walls attached to a black monitor screen (64 cm width × 3 cm depth, Liyama Prolite B2776HDS) and a metal bar floor with 1 cm spacings. A black polyoxymethylene strip with 8 reward wells (each 5 cm in height × 6 cm wide × 4 cm depth) was located below the screen, so that 0.1 mL 15% sucrose water rewards could be delivered from syringe pumps via plastic tubes. Identical visual cues were presented on the screen as white squares (2 cm width × 2 cm height, center‐to‐center stimulus distance = 6 cm) at 8 possible stimulus locations corresponding to the reward wells located below the screen. Stimuli were generated with Psychtoolbox‐3 in MATLAB (Brainard 1997) and the task setup was controlled with custom‐written scripts in MATLAB.

#### 2.2.1 Behavioral Pretraining

In the initial LD pretraining phase, animals learned to respond selectively and reliably to 1 out of 8 possible cued locations (L1–L8, see Figure 1A) and the rewarded location was randomly chosen out of those 8 sites every trial. At trial start, a green LED light turned on at the central well, after which the animal could initiate the trial with a nose poke into the central reward well for at least 0.3 s. After successful trial initiation, animals received a sucrose water reward with 20% probability to motivate the animal to self‐initiate trials. If the animal poked correctly at the cued location (“correct trial”), a sucrose water reward was immediately delivered at a reward well located below the corresponding cue stimulus. However, if the animal poked incorrectly, viz. at an uncued nose poke location (“incorrect trial”), a white LED light placed at the wall opposite the nose poke panel was lit for 3 s, followed by a 5 s time‐out. The visual cue disappeared after the choice was registered by a nose poke sensor located at each corresponding reward well. After the choice period, the trial ended, and an inter‐trial interval (ITI) of 10 s followed, during which the monitor screen was kept black to maximize the saliency of the white cue stimulus during trials. To proceed to the next phase of the experiment, the animal had to finish 90 trials within one session of 40 min with a performance of minimally 80% correct trials for two consecutive sessions.

#### 2.2.2 Viral Injection Surgery

After completing the pretraining, one batch of animals (*N* = 20 rats) was bilaterally injected in the dDG with pAAV5‐CaMKIIa‐hM4D(Gi)‐mCherry (virus titer ≥ 3 × 10^12^ vg/ml, Addgene, North Carolina, USA). Inhibitory DREADDs are activated by the artificial ligand clozapine‐N‐oxide (CNO; Hsiang et al. 2014; Roth 2016; Matos et al. 2019; Visser et al. 2020; Domi et al. 2021; Lesuis et al. 2021; but see Gomez et al. 2017) and transiently manipulate neural activity in targeted neurons for about 2 h following CNO injection (Roth 2016). Surgical procedures were performed in line with standard operating procedures for intracerebral viral injections. Animals received a subcutaneous injection of buprenorphine (0.01–0.05 mg/kg) and meloxicam (2 mg/kg) 20–30 min prior to anesthesia. Animals were anesthetized with isoflurane (induction level: 3%–5% isoflurane; maintenance level: 0.5%–3% isoflurane) and injected with 0.48 μL of the DREADD viral vector per injection site at a flow rate of 0.056 μL per min and 10 min post‐injection wait‐time. Injections were made with an automated nanoliter injector (Nanoject II, Drummond Scientific Company, USA) at the following anterior–posterior (AP), medial‐lateral (ML) and dorsal‐ventral (VL) coordinates relative to Bregma: −2.7 mm AP, ±1.2 mm ML, −3.7 mm DV in the anterior dorsal DG; −3.7 mm AP, ±2.0 mm ML, −3.1 mm DV in the posterior dorsal DG. For the spread of the virus in the DG, see section 3. After surgery, animals were solitarily housed for 3–7 days with soft food and water *ad libitum* provided.

#### 2.2.3 Behavioral Training

After recovery from viral injection surgery, animals were housed in pairs again and re‐trained on the pretraining paradigm until they reached the level of performance observed before surgery. Animals were then advanced to the training phase, in which they learned to discriminate between a rewarded and an unrewarded stimulus location with medium separation distance (18 cm). Animals initiated each trial similarly as in the pretraining phase, whereafter animals were presented with two visual cues and learned to identify which of the two locations was rewarded by means of trial‐and‐error. The animal received a sucrose water reward (0.1 mL, 15% sucrose in water) immediately upon poking if the choice was correct and a time‐out of 5 s if the choice was incorrect, cued by LED illumination of the training box (as described for the pretraining phase). After performing 9 out of 10 consecutive correct trials, the reward contingencies reversed to assess reversal learning and avoid a spatial bias to one side of the box (Figure 1B,C). Animals were trained for 6 sessions, during which the locations of each unique stimulus pair were varied over sessions with a consistent separation distance (18 cm per location pair, medium separation): location 1 and 3 (L1–L3), 2 and 4 (L2–L4), 3 and 5 (L3–L5), 4 and 6 (L4–L6), 5 and 7 (L5–L7), and 6 and 8 (L6–L8).

#### 2.2.4 Behavioral Testing With and Without DREADD Intervention

After completing training with medium distance, animals were ready for testing on the LD task with either small separation (L4–L5, distance = 6 cm) or large separation distance (L2–L7, distance = 30 cm) during neurointervention. Thirty to forty minutes before testing, animals received an i.p. injection with either the DREADD ligand clozapine‐N‐oxide (CNO; 3 mg/1 mL/kg in saline, HelloBio.com) or saline (vehicle, VEH; 1 mL/kg) as a control treatment. Animals were tested on each combination of task separation and neurointervention in a total of four sessions (one session per test day, Figure 1D): small separation with vehicle (henceforth abbreviated as Small‐VEH) or CNO (Small‐CNO) and large separation with vehicle (Large‐VEH) or CNO (Large‐CNO). To counterbalance the impact of task manipulations on LD performance, animals were divided into four cohorts that were each tested on a different task manipulation on each testing day (Figure 1D). Procedures for the LD test sessions were the same as described for the LD training sessions, with the only difference that the maximum number of reversals was set to 6 within 60 min, so that animals had a similar testing exposure across sessions.

### 2.3 Histology

After experiments had been completed, animals received a lethal dose of 1 mL 20% euthasol via an i.p. injection and were transcardially perfused with 4% paraformaldehyde (PFA) in phosphate buffered saline (PBS) to verify virus expression in the dorsal DG. Brains were extracted and stored in a 4% PFA solution at 4°C for 2 days. To prepare for slicing, brains were immersed in 15% sucrose in PBS for 1 day and 30% sucrose in PBS (pH 7.3) for 2 days. Brains were sliced into 40 μm coronal sections using a microtome and stored overnight in PBS at 4°C. To stain fluorescent cells tagged with mCherry, sections were stained with DAPI (300 nM in PBS, Sigma). Sections were immersed in DAPI for 5 min at room temperature, after which DAPI was removed and washed 3 times with PB (pH 7.3). Stained sections were mounted on microscope slides (SuperFrost Plus, Thermo Scientific) and stored overnight at room temperature. Sections were imaged using a fluorescence microscope (Leica Microsystems DM300) at 10× magnification to capture viral expression in the dDG (Figure 1E–G, Figure S1).

### 2.4 Data Analysis

#### 2.4.1 Location Discrimination and Reversal Performance

We first determined how task behavior and locomotion activity were modulated by stimulus distance and DREADD manipulation of the DG during the LD task.

Location discrimination performance was quantified by the following behavioral measures. The number of trials that the animals needed to reach the reversal criterion of 9 out of 10 consecutive correct choices was defined as the trials to criterion (TTC; Oomen et al. 2013; McTighe et al. 2009), where low and high values of TTC indicate fast and slow learning of the reward contingencies, respectively. The proportion of errors was computed as the number of incorrect choices divided by the number of total choices and indicates how well the animal discriminated between the target and nontarget cued locations. The response latency (in seconds) was defined as the time from stimulus onset until the animal poked at the target or non‐target location (L4 and L5 for Small sessions; L2 and L7 for Large sessions; Figure 1B). The number of blank pokes was defined as the number of nose pokes that were made into wells that were never cued nor rewarded during the test sessions (L1, L3, L6, and L8 for both Small and Large sessions; Figure 1B). The number of blank pokes (i.e., at noncued wells) was determined to probe how spatially precise animals poked at the target and nontarget wells.

Each task performance measure was compared between the task separation and treatment conditions for each learning phase. Measures were separately computed for each learning phase to contrast task performance across different learning phases, viz. the acquisition phase (Acq, where the animal did not adjust its behavior to a first reversal yet) and the subsequent reversal phase, where the temporal rank order of reversals is indicated by *R*_1_, *R*_2_, …, *R*_*n*_. We fitted a generalized linear mixed model (GLMM) to each behavioral measure to account for the crossed random effects of the data (Equation 1; function *fit_glm*, MATLAB), since the model factors treatment and separation were crossed within each animal. Each GLMM was fitted with a set of distributions, including the normal, inverse Gaussian, Poisson, and gamma distribution, and the best model fit was determined as the model with the lowest sum of squared errors (SSE). We then performed an ANOVA on the model coefficients to test for the main and interaction effects of treatment and separation. Post hoc comparisons between the treatment conditions for each separation were made with a Wald test on the estimated marginal means (function *emmeans*, *MATLAB*, source code: github.com/jackatta/estimated‐marginal‐means), computed as the means of each model factor adjusted for the means of the other model factors. All post hoc *p* values were adjusted with a false discovery rate correction for multiple comparisons. The significance level was set at *p* < 0.05.

The GLMM was specified in general form as follows:

(1) $$Y_{i}=\beta_{0}+\prod \beta_{p}\;X_{\mathrm{pi}}+\prod \upsilon_{p}\;Z_{\mathrm{pi}}+\varepsilon_{i}$$

here, $Y_{i}$ is the behavioral task measure corresponding to the levels of the predictor variables $X_{1i}$, $X_{2i}$, …, $X_{\mathrm{pi}}$ and random effects variables $Z_{1i}$, $Z_{2i}$, …, $Z_{\mathrm{pi}}$ of each trial *i*. The beta coefficients $\beta_{0}$, $\beta_{1}$, …, $\beta_{p}$ correspond to the predictor variables $X_{\mathrm{pi}}$, where $\beta_{0}$ is the intercept. The random effects coefficients $\upsilon_{p}$ correspond to the random effect variables $Z_{\mathrm{pi}}$. Lastly, $\varepsilon_{i}$ indicates the residual error of the model fit for each trial *i*. The product terms of the predictor and random effects variables indicate the interaction term between the task variables separation × treatment. The weights of the coefficients are not described in the current study, as the coefficients differed for every model fitted, task learning phase and behavioral task measure.

#### 2.4.2 Locomotion Activity During Task Behavior

To control for non‐specific DREADD effects on locomotion, we also quantified the rat's body position from video data collected at 25 fps for a subset of the animals showing bilateral DREADD expression (*N* = 7). Video frames were processed with DeepLabCut (Mathis et al. 2018), a semi‐automated tracking program that uses deep neural networks to estimate the position of body parts based on manually labeled test frames. We labeled 6 body parts every 80–120 frames per animal, including the body center, left shoulder, right shoulder, neck base, and snout. To obtain a reliable estimate of body position, we averaged the body part coordinates to a center‐of‐mass (*X*, *Y*) coordinate for every frame. Noise artifacts were reduced by removing those estimated coordinates that fell outside of the LD box, whereafter the tracking data was smoothed with a moving average filter (width: 10 frame samples) (function *smoothdata*, MATLAB) and an interpolation filter was applied to a window of 100 msec (function *interp1*, MATLAB). Locomotion activity was quantified as body speed, defined as the traveled distance per second (cm/s) from trial start until trial end. The traveled distance was computed as the square root of the sum of squared differences between each pair of *X* coordinates and squared differences between each pair of *Y* coordinates. Body speed was compared between the four different combinations of separation and treatment conditions with a GLMM, separately for each reversal. Post hoc comparisons between treatment conditions were made with estimated marginal means.

#### 2.4.3 Fitting Choice Data to Reinforcement Learning Models

To understand which aspects of learning during spatial discrimination were affected by DREADDs, we estimated the learning parameters underlying place‐outcome learning driven by reward using a reinforcement learning model (henceforth referred to as “RL model”; Rescorla and Wagner 1972; O'Reilly and den Ouden 2015; Sutton and Barto 2018; Metha et al. 2020). The RL model was fitted to choice behavior to track trial‐to‐trial changes in reward‐driven learning over time. This allowed us to determine whether the DG‐dependent spatial disambiguation deficit observed in previous studies (Gilbert et al. 2001; Hunsaker and Kesner 2008; Morris et al. 2012; Lee and Solivan 2010) could be explained by a deficit in acquiring place‐outcome associations. We thus applied a behavioral model to probe learning parameters of behavior.

**FIGURE 2:**
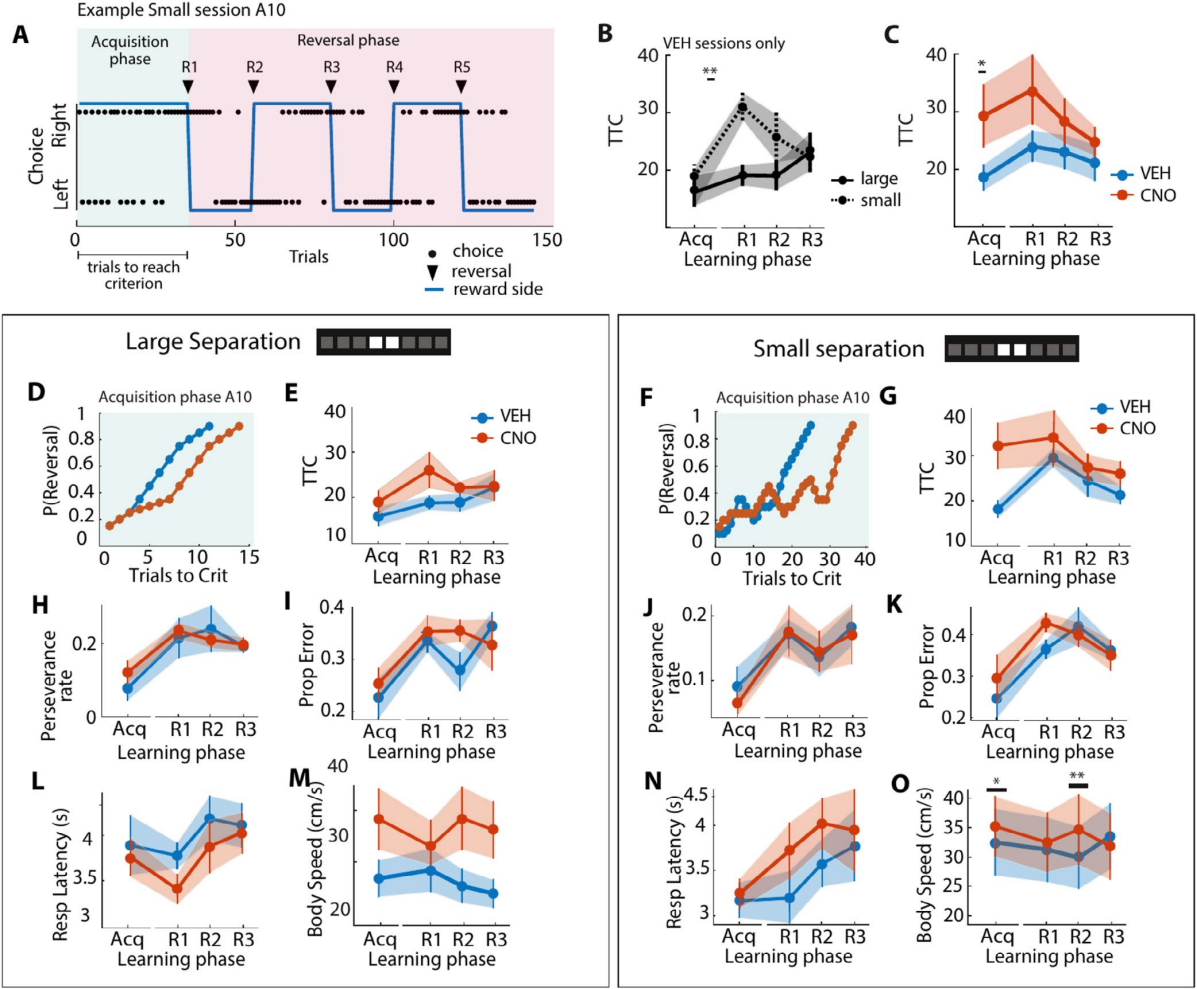
Task behavior during location discrimination task for CNO and control treatment conditions. (A) Example choice behavior for a representative animal (A10) during a Small session with CNO treatment. Black dots indicate left‐ and right‐ward choices. The blue line indicates the rewarded side (left or right). Black solid triangles indicate when the reversal criterion was reached and reward contingencies reversed (reversal 1 as R1, reversal 2 as R2, etc.). (B) Trials to reach criterion (TTC) for baseline (VEH) treatment across Large (solid line) and Small (dotted line) sessions (*N* = 11). (C) TTC pooled across Small and Large sessions for animals with VEH (blue line) and CNO (orange line) treatment for each learning phase. In all subsequent panels, left‐side panels represent Large sessions (panels D, E, H, I, L, M) and right‐side panels represent Small sessions (panels F, G, J, K, N, O). (D, F) Example session showing the cumulative reversal probability across trials for the same animal (A10) as panel A with saline (VEH, blue line) and CNO treatment (orange line) during the acquisition phase. (E, G) TTC with VEH (blue line) and CNO (orange line) treatment for each learning phase. (H, J) Proportion of trials where the same choice was made as the previous trial (perseverance rate) for each learning phase. (I, K) Proportion of incorrect trials for each learning phase during Large (panel I) and Small (panel K) sessions. (L, N) Response latency (in sec) for each learning phase. (M, O) Body speed (in cm/s) during the choice epoch for each learning phase. Error bars indicate average task variables pooled across animals (mean ± SEM). Significance is indicated by *p < 0.05, **p < 0.01.

Specifically, the RL model allowed us to investigate whether the learning of place‐outcome associations was driven by reward, viz. by assessing the learning rate (*alpha* parameter) and the tendency of animals to choose the rewarded location based on the reward value associated with each place (*beta* parameter). In turn, the quantification of these reward‐related parameters may explain why we observed a selective dDG‐driven deficit during the acquisition phase of the task (Figure 2D,F), when animals presumably acquired place‐outcome associations. Moreover, to determine whether animals persisted in their choice despite receiving reward in the previous trial, we extended the RL model with an additional perseverance parameter (*delta* parameter; henceforth referred to as Perseverance RL model, “PRL model”; adjusted from Metha et al. 2020).

To model how the choice behavior of animals during the LD task was modulated by stimulus distance and DREADD manipulation of the DG, we fitted the choice data of each animal to the RL and PRL models. The models were then compared using the Akaike Information Criterion (AIC; Akaike 1998) to determine which best fit the behavior of each animal. We first fitted the RL model to the choice data (Rescorla and Wagner 1972; Metha et al. 2020; Sutton and Barto 2018). This model was structured as follows. For each trial *t*, the probability of choosing the right stimulus ($P_{\text{right},t}$) was computed with a sigmoid function (Equation 2) that models how the learned stimulus values ($V_{\text{right},t}$ and $V_{\text{left},t}$) translated into a choice (Equation 3). The sigmoid equation generally follows the behavior observed in humans and rodents performing reversal tasks (O'Reilly and den Ouden 2015) where the subject is more likely to choose the option with the higher stimulus value, but can also switch to the less valued choice from time to time by way of spontaneous alternation. The choice probability thus remains stochastic, that is without reaching values of 0 or 1. The value of each stimulus ($V_{\text{right},t}$ and $V_{\text{left},t}$) was modeled with the Rescorla–Wagner equation (Rescorla and Wagner 1972) that follows prediction‐error based learning, in which the stimulus gains reward value when the outcome on the previous trial ($r_{t-1}$) exceeds the predicted reward value on the previous trial ($V_{t-1}$). Importantly, the prediction error scales with the learning rate *alpha*, that represents how quickly animals acquire the reward value when the stimulus value changes across trials (e.g., a low *alpha* indicates slow learning). We then estimated the sensitivity to reward with the slope parameter *beta*, that indicates how strongly the (binary) choice of the animal follows the stimulus value (graded) across trials (e.g., a low *beta* indicates that the animal chooses closer to chance level, rather than following stimulus value). For the Perseverance RL (PRL) model, the RL model was extended with a *delta* parameter that estimates the degree with which the animals persisted in the choice of the previous trial (Equation 4). A positive value of *delta* indicates that an animal was more likely to respond on the same side as the previous trial (perseverance). Conversely, a negative value indicates that animals were more likely to switch from side to side on consecutive trials (alternation). In this model (Equation 4) $C_{\text{left}}$ and $C_{\text{right}}$ are indicator variables, taking the value of 1 if the corresponding stimulus is chosen, and 0 otherwise. The parameters *alpha*, *beta*, and *delta* were estimated for each animal based on the maximum likelihood fit to the choice data. We then compared the *alpha*, *beta*, and *delta* parameters between treatment conditions for every separation condition with a GLMM as specified in Section 2.4.1. All models were fitted with a constant offset value of 0.5 to reflect the choice probability at chance level at the start of each session. *p* values were adjusted with an FDR correction for multiple comparisons. Significance levels were set at *p* < 0.05.

(2) $$P_{\text{right},t}=0.5+\frac{1}{1+e^{-\beta \left(V_{\text{right},t}-V_{\text{left},t}\right)}}$$

(3) $$V_{\text{right},t}=V_{\text{right},t-1}+\alpha \left(r_{t-1}-V_{\text{right},t-1}\right)$$

(4) $$P_{\text{right},t}=0.5+\frac{1}{1+e^{-\beta \left(V_{\text{right},t}-V_{\text{left},t}\right)+\delta \left(C_{\text{left}}\left(t-1\right)-C_{\text{right}}\left(t-1\right)\right)}}$$

## 3 Results

To study the role of the dorsal dentate gyrus (dDG) in disambiguating similar spatial representations, we selectively modulated the dDG with an inhibitory DREADD (AAV5‐hM4D(Gi)‐mCherry) in Lister Hooded rats (*N* = 20) that performed a location discrimination (LD) task (Oomen et al. 2013). In this task, animals discriminated between a rewarded and an unrewarded location as indicated by a CS+ and a CS− visual cue (Figure 1A), where the separation distance was either small (i.e., similar location) or large (i.e., dissimilar location). Within each session, reward contingencies were switched after 9 out of 10 correct consecutive choices (“reversal criterion”; Figure 1B,C) to avoid spatial biases for either cued location and to allow us to probe the spatial discrimination ability. Animals were injected with either saline (vehicle treatment, VEH) or CNO (test treatment) and tested in each separation condition in a counterbalanced manner.

Upon histological inspection, we first included 11 out of 20 animals for behavioral analysis, based on whether we observed bilateral DREADD‐mCherry expression in the left and right DG (Figure 1E–G). We identified DREADD+ transduction in the dDG based on mCherry expression in the hilus, granule cell layer, and mossy fiber pathway along the anterior–posterior axis. For each DG subregion, we qualitatively assessed if there was mCherry expression in the left and right hemisphere (Goossens et al. 2021; Shen et al. 2021). Animals were included for further analysis if they showed virus expression in at least two subregions in both left and right DG. This resulted in the exclusion of six animals with unilateral virus expression in the dDG and three animals with no virus expression in left nor right dDG. Animals that were included based on this criterium showed virus expression patterns (Figure S1) very similar to studies targeting the DG with hM4Di‐mCherry (Panoz‐Brown et al. 2018; Shen et al. 2021) and hM3Dq (Keenan et al. 2017), with predominant labelling of the granule cell layer, hilus, and mossy fiber projections terminating in the CA3. Specifically, mCherry was clearly expressed in cell bodies in the granule cell layer and hilus subregion, while dense axonal projections were observed from the DG onto CA3 (Figures 1G and S1A,B; Panoz‐Brown et al. 2018; Shen et al. 2021). mCherry expression was observed in the dorsal part of the DG, extending from −1.72 mm to −4.56 mm AP from Bregma (Figures S1 and S2). To determine whether individual viral injections were successful, we inspected mCherry expression near the anterior (−2.77 mm AP, ±1.2 mm ML, −3.77 mm DV) and posterior injection sites (−3.7 mm AP, ±2.0 mm ML, −3.1 mm DV; see Section 2.2.2). We observed expression in both hemispheres and at both injection sites in all rats (approximately between 1.72 and −4.56 AP; Figure S2A–J), with the exception of rat 11. Rat 11 did not show clear expression at the posterior right injection site (Figure S2K), suggesting that the viral injection at this site likely failed. Thus, based on the spread of virus expression limited to the dorsal DG, we consider it unlikely that the posterior DG (extending beyond −4.56 mm AP from Bregma) was targeted.

Apart from the DG, we observed dense localized staining in the CA3 characterized by stripy structures without cell bodies, likely indicating mossy fiber projections from the DG terminating in the CA3 (Panoz‐Brown et al. 2018; Shen et al. 2021). We also observed mCherry expression in individual cell bodies to a lesser degree (Figure S1), though this was difficult to assess due to the dense spread of stripy structures. Localized staining in the CA3 in both hemispheres was observed in every rat, that is, rats with DREADD+ expression in the dDG always co‐expressed mCherry in the CA3, which is very similar to virus expression patterns from previous studies targeting the DG (Shen et al. 2021). We also observed mCherry expression in the CA1 layer bilaterally in 3 out of 11 rats (27%, Figure S1A) and unilaterally in 4 out of 11 rats (36%, Figure S1B), likely because the CA1 layer had to be penetrated to have the injections reach into the DG. mCherry expression was also observed bilaterally and unilaterally in the subiculum layer in 3 out of 11 (27%) and 5 out of 11 rats (45%). We observed qualitatively similar task behavior in rats co‐expressing DREADDs in the CA3, CA1, and subiculum bilaterally (data not shown) and therefore included these rats for further analysis. Together, DREADD‐mCherry was mainly expressed in the dorsal part of the DG, but a co‐involvement of areas CA3, CA1, and subiculum cannot be excluded (see Section 4.1).

### 3.1 Transient DREADD Manipulation of Dorsal DG Cells Impairs Spatial Discrimination During Task Acquisition

We next determined how spatial disambiguation learning was affected by inhibitory DREADD modulation of the dDG in the subset of animals with bilateral DREADD+ expression (*N* = 11). Task performance was probed as the number of trials needed to reach the reversal criterion (trials to criterion, TTC; Clelland et al. 2009; McTighe et al. 2009; Oomen et al. 2013). Importantly, to track the temporal engagement of the dDG during discrimination learning within a session, we separately probed task performance for the acquisition phase and subsequent reversal phases (Figure 2A) for each treatment and separation condition. On average, animals made 6 reversals (± 0.10 SEM) for Large sessions and 5 reversals (±0.21 SEM) for Small sessions (Figure S3A). There was a main effect of separation on the number of reversals made by DREADD+ rats (*F*(40) = 39.61, *p* = 1.82E‐07, GLMM), indicating that animals made fewer reversals when discrimination was more difficult. No effect of treatment was observed on the number of reversals (*F*(40) = 1.10, *p* = 0.30, GLMM), showing that animals remained able to perform the reversal task rule in spite of chemogenetic manipulations.

To determine whether task performance depended on the separation distance, we first compared TTC between Small and Large sessions for each task phase for vehicle sessions only (see Table S1). There was a main effect of separation on task performance after the first reversal was made (*F*(19) = 11.60, *p* = 0.0030, GLMM; Figure 2B), showing that animals performed worse in Small compared to Large sessions. No effect of separation distance was observed for the acquisition phase and subsequent reversals within the same session (*p* > 0.05, GLMM; Figure 2B; Figure S3A). These findings show that animals performed worse after the first reversal but rapidly improved their performance and reached similar performance levels within the same session regardless of discrimination difficulty.

We next determined how the task performance was modulated by the treatment depending on the separation distance, where the TTC was compared between treatment sessions for each separation level and task phase separately (Figure 2A; see Table S1). For the acquisition phase, there was a main effect of separation (*F*(40) = 5.49, *p* = 0.024, GLMM; Figure S3C) and treatment on TTC (*F*(40) = 5.75, *p* = 0.021; Figure 2C; Figure S3D), but no interaction effect of these factors (*F*(40) = 2.91, *p* = 0.096; Table S1). Although we observed a trend that animals performed worse when CNO was administered for similar stimulus locations (Figures 2F,G and S3F), we did not perform post hoc tests to support this observation due to the absence of an interaction effect. The treatment‐dependent task impairment during the acquisition phase thus did not depend on the task difficulty. In contrast, we did not observe an effect of treatment on the TTC for any reversal (*p* > 0.05, GLMM; Figures 2C and S3C). Although there was a main effect of separation after the animal made its first reversal (*F*(38) = 11.89, *p* = 0.014; Figure S3C), there were no interaction effects during any reversals and no post hoc tests were carried out as a result (*p* > 0.05, GLMM; Figures 2D–G and S3E,F). Together, our findings show a DG‐dependent discrimination deficit during the initial acquisition phase that recovers to baseline performance levels within the same session.

To elaborate on the observed variability in task performance between animals after CNO treatment (Figure 2C), we separately visualized the TTC for each rat across learning phases (Figure S4A–K). During the acquisition phase, we observed that task performance was impaired after CNO treatment in 6 rats (Figures S4B,C, S4F, S4H, S4J,K). The remaining rats showed either no effect of treatment (4 rats, Figures S4A,D,E and S4G) or a slightly improved task performance (1 rat, Figure S4I). Moreover, we determined how task behavior was affected by variability in virus expression. The majority of rats showed impaired task performance under CNO treatment across multiple reversals (*N* = 9; Figures S3A–D, S3F,G, S3I–K), in line with the treatment effect at the group level that we reported (Figure 2C). Importantly, rat 11 (not displaying a clear expression at the posterior injection site in the right hemisphere) also showed a strong effect of CNO during the acquisition phase. Although it is difficult to speculate based on one animal, we conclude that DREADD expression in the posterior part of the DG does not explain the effect of CNO on acquisition learning. Moreover, to determine whether there was a confounding effect of testing day number on task performance, we fitted a GLMM to TTC with the model factors of treatment, separation, and testing day number, as described in the Methods (Section 2.4.1). We then performed an ANOVA on the model coefficients to test for the main and interaction effects of treatment, separation, and testing day number. When considering both the acquisition phase and the subsequent reversals, we observed a main effect of treatment (*F*(36) = 4.31, *p* = 0.039) and testing day number (*F*(36) = 7.69, *p* = 0.006), but did not observe any interaction effects (*p* > 0.05). We also performed the same analysis only on the acquisition phase; however, the inclusion of testing day as a fixed factor lowered the power of our analysis and no main nor interaction effect turned out to be significant. To account for the reduced power, and considering the consistent non‐significant results for any interaction factor, we repeated the analysis including only main effects and, consistently with the previous results, we observed a main effect for treatment (*F*(39) = 4.47, *p* = 0.043) and separation (*F*(39) = 7.64, *p* = 0.010), but only an almost significant trend for testing day (*F*(39) = 3.965, *p* = 0.056). Specifically, we observed that task performance in the acquisition phase slightly improved across testing days (Figure S3G). Nevertheless, the absence of an interaction effect suggested that the CNO‐dependent task impairment did not depend on the testing day number.

To determine whether other measures of task performance were affected by modulating the dDG, we also compared the proportion of incorrect choices (error proportion, inverse of proportion correct; Gilbert et al. 2001; Lee and Solivan 2010; Oomen et al. 2013, 2015), the time needed to report the choice (response latency; Oomen et al. 2013), the tendency of the animal to persevere in its previous choice (perseverance rate) and the spatial accuracy of reported choices (see Table S1). There was no effect of treatment on these performance measures for each separation level and task phase separately (*p* > 0.05, GLMM and Wald test; Figure 2H–L, Figure 2N; Figure S5). Thus, there was no effect of dDG manipulation on the animal’s ability to report its choices during the task. Finally, to probe the spatial accuracy of choices, we quantified the response distributions for target (cued) and non‐target (noncued, i.e., blank) locations. The spatial distributions of nose pokes revealed no difference between the treatment conditions regardless of the separation distance and task phase (see Table S1; *p* > 0.05, Kolmogorov Smirnov test; Figure S6A,B). These findings suggest that dDG inactivation did not impair the animal's ability to locate the cued stimulus locations in space. Together, our findings indicate that CNO did not affect an animal's ability to report its choices during the discrimination task, nor the ability to spatially locate the cued target locations, except for the CNO effect on the ability to consecutively and correctly respond to the rewarded side (Figures 2C and S4C).

We next examined the possibility that the observed discrimination deficit (Figure 2C) could be caused by side effects of CNO being metabolized into clozapine on locomotion behavior (Gomez et al. 2017; MacLaren et al. 2016). We quantified the movement speed in a subset of animals during task engagement and for which we could obtain reliable motion tracking across entire recording sessions (*N* = 7; Figure S6C,E; see Table S1). There was an interaction effect between separation and treatment on body speed during the acquisition phase (*F*(28) = 5.49, *p* = 0.027, GLMM), where animals moved significantly faster for CNO compared to VEH treatment during the acquisition phase (*W* = 2.58, *p* = 0.031, Wald test) and after the second reversal of Small sessions (*W* = 3.27, *p* = 0.0063, Wald test; Figure 2O; Figure S6F). Interestingly, this task‐phase and treatment‐dependent effect on body speed was observed selectively when animals made correct (Figure S6I) and leftward choices (Figure S6M). In contrast, no effect of CNO on body speed was observed during Large sessions (Figure 2L; Figure S6D), independent of the correctness of the trial (Figure S6G,H) and of whether a response was made to the left or right side (Figure S6K,L). Thus, the effect of CNO on body speed did not generalize across separation levels, suggesting that there was no general effect of CNO administration on locomotion activity.

### 3.2 Increased Choice Alternation Underlies Impaired Discrimination Learning During DG Modulation

The dDG‐dependent discrimination deficit observed during the acquisition phase (Figure 2C) was characterized by an increased number of trials needed to reach performance criterion. In other words, animals showed an initial impaired ability to make consecutive correct choices, indicative of slower acquisition learning across trials. An alternative interpretation could be that animals alternate more often between choices as a strategy to maximize reward output when spatial discrimination is ambiguous. For example, animals could have trouble identifying the rewarded side when place‐outcome associations have not yet been established, that is during the acquisition learning phase. To discern between these two interpretations, we set out to capture place‐outcome learning dynamics and the volatility of choice behavior across trials with two variants of a reinforcement learning (RL) model suitable to the behavioral task at hand (Rescorla and Wagner 1972; Sutton and Barto 2018; Metha et al. 2020). The first RL model that we used (henceforth: “Simple RL model”) characterized learning dynamics with two parameters. First, the learning rate (*alpha*) indicates how quickly animals adapt their choice across trials and reflect the association strength between location and reward. Second, the reward sensitivity (*beta*) indicates how sensitive animals are to differences in reward value. The second RL model (henceforth: “Perseverance RL – PRL – model”; Metha et al. 2020) included an additional parameter besides the learning rate and reward sensitivity: the perseverance rate (*delta*), indicating the tendency of animals to persevere or alternate in their choice behavior.

**FIGURE 3:**
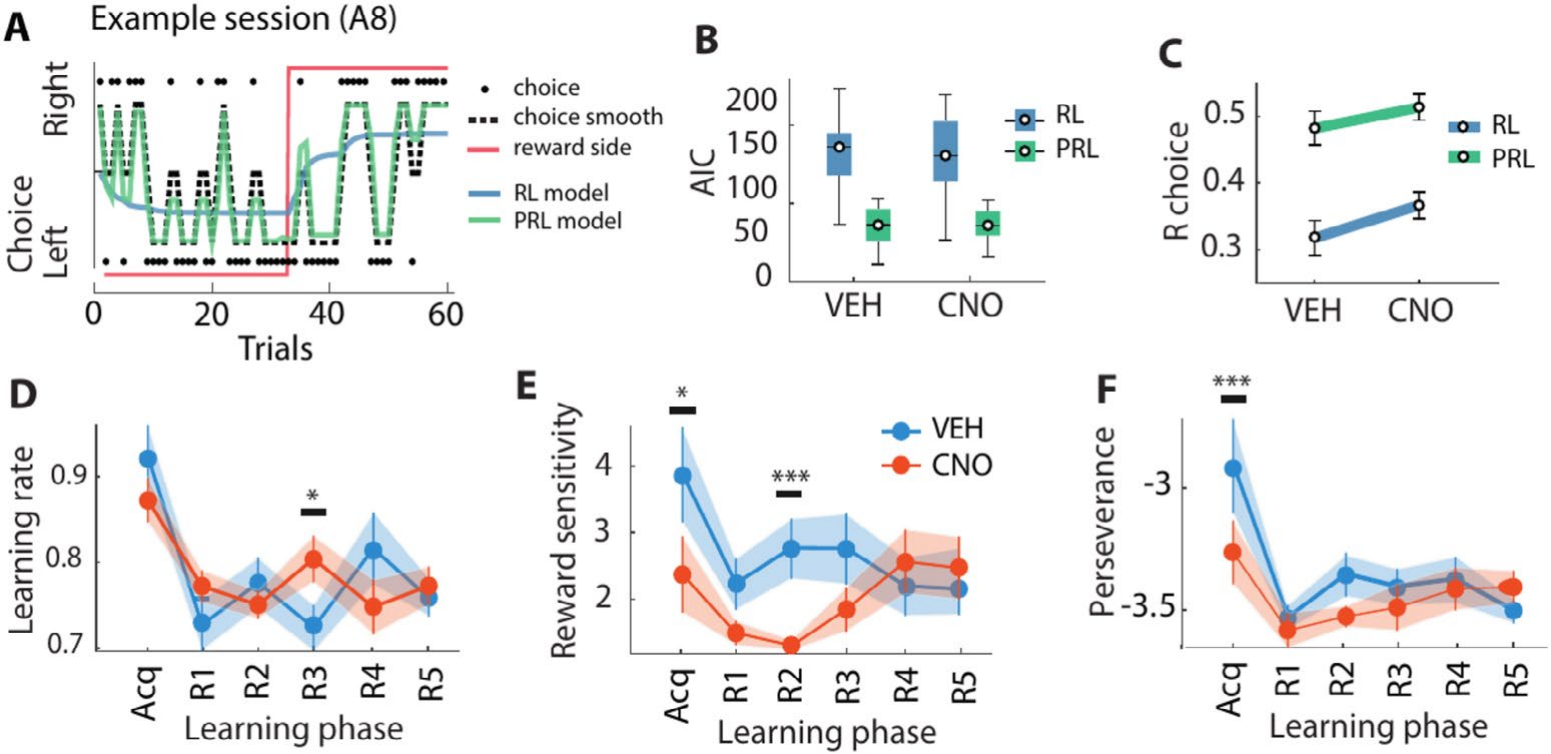
Increased choice switching associated with bilateral DG‐dependent discrimination deficit during. Acquisition learning of place reward associations. (A) Representative example session of choice behavior per trial (black dots) and smoothened across 4 trials (back dotted line) of a representative animal 8 (A8) during a Small separation session with CNO treatment. The choice trace is overlaid with the modeled behavior from the simple reinforcement learning (RL) model (blue line) and the perseverance RL (PRL) model (green line). The rewarded side is indicated with the red line (reward side switches after the animal made 9 out of 10 consecutive correct trials). (B) Median akaike information criterion (AIC) of the RL (blue square) and PRL model fits (green square) per treatment condition. (C) Per‐session correlations (R) between the observed and modeled choices per treatment condition as fitted by the RL (blue line) and PRL model (green line). (D) Median learning rate (alpha) for each learning phase with saline (VEH, blue line) or CNO (orange line) treatment. (E) Same as panel D, but for the median reward sensitivity (beta). (F) Same as panel D, but for the median perseverance rate (delta). Boxplots indicate the interquartile range (IQR) of task variables pooled across animals (median ± IQR). Error bars indicate average task variables pooled across animals (mean ± SEM). Significance is indicated by *p < 0.05, ***p < 0.001.

We fitted the RL models for each individual animal, task condition, and learning phase on a trial‐by‐trial basis (Figures 3A and S7A), which allowed us to obtain the parameter estimates separately for each rat per task condition and learning phase. The PRL model returned a lower Akaike Information Criterion (AIC) value compared to the simple RL model for every model fit for each animal (*N* = 11, Figures 3B and S7B). Given that a lower AIC value indicates a significantly better model fit, these findings suggested that the variability in choice behavior was better captured if we took the tendency of animals to persist in their choices into account. Moreover, the trial‐by‐trial estimates of the PRL model predictions correlated significantly stronger with the observed animals' choice behavior compared to the simple RL models (Figure 3C and S7C). Our findings thus indicate that the Perseverance RL model is a better fit of choice behavior compared to the Simple RL model.

We next compared the best fit parameter values across conditions for the PRL model (see Table S1). In the PRL model, we observed no main effect of treatment (Figure 3D) or separation (Figure S7D) on the learning rate parameter (*alpha*) for any task phase (*p* > 0.05, GLMM; Table S1), with the exception that the learning rate was higher for CNO compared to vehicle sessions after the third reversal was made (*F*(35) = 7.13, *p* = 0.011; Figure 3D). In comparison, the reward sensitivity parameter (*beta*) was significantly reduced upon CNO treatment during the acquisition phase (*F*(40) = 4.34, *p* = 0.044) and after the second reversal was made (*F*(36) = 16.18, *p* = 2.80E‐4; Figure 3E). There was no effect of separation on reward sensitivity for any task phase (*p* > 0.05, GLMM; Figure S7E). Finally, the perseverance parameter (*delta*) was significantly lower after CNO compared to vehicle treatment during the acquisition phase (*F*(40) = 64.54, *p* = 7.11E‐10; Figure 3F). The lower perseverance value indicated a higher tendency to alternate between choices upon CNO treatment. We found no effect of separation on the perseverance at any task phase (*p* > 0.05, GLMM; Figure S7F). Together, the PRL model revealed that a reduced reward sensitivity and enhanced choice alternation were temporally associated with the dDG‐dependent spatial discrimination deficit during the acquisition phase, with no relation to separation distance.

## 4 Discussion

The main goal of the present study was to investigate the acute and causal role of the dorsal DG (dDG) in disambiguating similar spatial stimuli. We found that a temporary, DREADDs‐mediated dysfunction of the dDG during a location discrimination task causes a transient discrimination deficit during task acquisition (Figure 2C). This discrimination deficit did not depend on separation distance, indicating that the effect we observed applied to the discrimination of both similar and dissimilar spatial locations. Our study thus suggests a role of the dDG in the learning of place‐reward associations when animals perform a location discrimination task, as shown by two novel findings. First, task performance rapidly recovered to baseline levels after the acquisition phase (Figure 2C), indicating that the dDG was selectively engaged during the initial formation of place‐reward associations. Second, our modeling study revealed that this dDG‐dependent deficit was associated with a decreased reward sensitivity (Figure 3E) and increased tendency to alternate between choices across trials (Figure 3F). Taken together, our study provides novel evidence showing that, when chemogenetics—rather than permanent lesioning—is used, the dDG is acutely but transiently engaged in the acquisition learning of place‐outcome associations.

### 4.1 Transient Bilateral Modulation of the Dorsal DG Causes Spatial Discrimination Deficits—Chemogenetics Versus Permanent Lesions

Our finding that the discrimination of similar, as opposed to dissimilar, spatial stimuli did not rely on the dDG was based on the absent interaction effect between separation and treatment on TTC (Table S1). However, the observed variability in virus expression (Figure S2) and task performance (Figure S4) between animals likely decreased the power of our statistical model. In turn, this may explain the discrepancy between our study and previous lesion studies that showed separation‐dependent effects upon dDG dysfunction (Gilbert et al. 2001; Hunsaker and Kesner 2008; Morris et al. 2012; Lee and Solivan 2010). In support of this, we observed a nonsignificant trend towards interaction between treatment and separation distance on TTC (Table S1), pointing towards a possible role of the dDG in discriminating similar spatial stimuli.

Nevertheless, our study challenges the widely held view of the dDG in fine‐grained spatial disambiguation as suggested by previous lesion (Gilbert et al. 2001; Hunsaker and Kesner 2008; Morris et al. 2012; Lee and Solivan 2010) and gene knock‐out studies (McHugh et al. 2007; Kannangara et al. 2015; Yun et al. 2023; but see Oomen et al. 2015) in rodents. A possible explanation for this discrepancy could be that the DG is more sensitive to dysfunction after lesioning compared to chemogenetics. Indeed, toxins such as colchicine and ibotenic acid have been shown to inflict permanent damage and loss‐of‐function within this subregion (Gilbert et al. 2001; Lee and Kesner 2003; Lee and Kesner 2004; Jerman et al. 2005). Previous studies have shown that permanent tissue damage affects downstream areas within the neural circuit in a cascade‐like manner (Otchy et al. 2015; Vaidya et al. 2019) or causes compensation of affected functions via other areas within the functional circuit (Zelikowsky et al. 2013; Hong et al. 2018; reviewed in Vaidya et al. 2019). In such a case, dysfunction in the neural circuit supporting discrimination learning may be more severe because it not only concerns the DG, but also its downstream areas such as the CA3 and CA1. Indeed, lesioning of granule cells in the DG with colchicine in rats discriminating between visual scenes has been shown to not only impair behavioral performance, but also degrade the ability of downstream CA3 cells to discriminate between ambiguous scenes by scene‐dependent firing rate modulation (Lee and Lee 2020). In comparison, our study used reversible DREADDs to transiently modulate the firing activity of excitatory cells in the DG, making it more plausible that the functional circuit was preserved despite chemogenetic manipulation, resulting in more subtle discrimination‐related deficits.

An alternative, yet complementary explanation could be related to our observation that the density of DREADD‐mCherry+ expression in the target area dDG varied between animals upon histological verification (Figure S1). In line with this, we observed large variability in task performance across individual rats treated with CNO as compared to saline treatment (Figure 2C). Moreover, we observed co‐expression of DREADD‐mCherry+ in the CA3, CA1, and subiculum in a substantial subset of our animals (see Results, Figure S1A). This may have affected the behavioral performance differentially, making it difficult to estimate to what extent co‐expression in these subregions impacted task performance in our study. One previous study showed that the lesioning of the CA3 and CA1 did not impair the ability to discriminate between adjacent locations in rats (Gilbert et al. 2001), arguing against the possibility that co‐targeting of these subregions impacted task performance in our study. Contrary to this, a large body of studies has proposed that the neural circuit supporting disambiguation learning not only involves the DG, but also the CA3 (GoodSmith et al. 2019; Lee et al. 2015; Lee and Lee 2020; Leutgeb et al. 2007; reviewed in Rolls 2013) onto which the DG projects densely via the mossy fibers (reviewed in Amaral and Witter 1989). Given that the CA1 and subiculum are typically not implicated in disambiguation learning, we speculate that co‐expression in the CA3 may have partially confounded the DG‐dependent treatment effect on spatial discrimination observed in our study.

Aside from off‐target and compensation effects impacting task performance, previous studies have also shown that lesioning the DG induces hyperactivity (Barone Jr et al. 1992; Emerich and Walsh 1990). To examine whether there was a confounding effect of motor movement after chemogenetic targeting of the DG, we quantified the body speed during task engagement. Animals tended to move faster, but only during the acquisition phase and after the second reversal of Small sessions under CNO (Figures 2N and S6F), and specifically in correct (Figure S6I) and leftward trials (Figure S6M). These findings suggest that there was no general effect of chemogenetics on locomotion during the task. We thus presume that the hyperactivity observed during acquisition learning (Figure 2O) was a correlate of the behavioral impairment experienced by animals following temporary dysfunction of the DG (Figure 2C).

### 4.2 Spatial Discrimination Learning: Working Memory, Place‐Outcome Learning, or Spatial Learning

To understand which aspects of spatial learning were affected by dDG dysfunction, we separately assessed discrimination learning during the acquisition phase and subsequent reversal phase (Figure 1C), as well as the learning dynamics underlying place‐outcome learning using a reinforcement learning model (O'Reilly and den Ouden 2015; Metha et al. 2020). The transient causal engagement of the dDG only during the acquisition phase (Figure 2C) renders it unlikely that the discrimination deficit can be explained as a general working memory or perceptual impairment, as such a deficit would persist across reversals. We observed no response bias for left or right separated cued locations (Figure S5G,H), suggesting that spatial response learning based on an egocentric reference frame was also intact in these animals (e.g., the reward location is at the left side of the animal and not at its right). This agrees with earlier findings that this type of response learning is hippocampus independent and relies instead on the dorsal striatum (Packard and McGaugh 1996; Featherstone and McDonald 2004).

Instead, the selective, causal engagement of the dDG only concerned the initial learning of place‐reward associations (Figure 2C), pointing to a deficit in learning to discriminate rewarded versus nonrewarded locations (McDonald and White 1995; Gilbert et al. 2001; Hunsaker and Kesner 2008; McHugh et al. 2007; Morris et al. 2012; Lee and Solivan 2010; Kannangara et al. 2015; Yun et al. 2023). Specifically, the impaired ability to respond consecutively and correctly to the target location despite receiving reward hints towards a learning or spatial deficit: either a deficit in learning which place was associated with reward or, alternatively, a deficit in spatially separating stimulus locations. We also observed no differences in the spatial distribution of blank pokes surrounding the target locations between treatment sessions (Figure S6A,B), suggesting that animals with dDG dysfunction did not respond less spatially precisely when CNO was administered compared to control treatment sessions. Moreover, the dDG was engaged during the acquisition phase regardless of the spatial separation (Figure 2C), also arguing against a spatial deficit. Instead, the observation that the sensitivity of choice behavior to differences in reward value was reduced following dDG inactivation during the acquisition phase (Figure 3E) hints toward a place‐reward learning deficit. Moreover, animals responded less consistently to the rewarded stimulus across consecutive trials (Figure 2C) and alternated more frequently (Figure 3F). This suggests that dDG dysfunction causes a deterioration both in the ability to associate rewards to locations and in the stability of choice behavior. The effects that we observed point towards a role for the dDG in learning or maintaining correct place‐reward representations during the acquisition of place‐outcome associations, i.e., learning which location yields a reward. Volatile choice switching is typically associated with dysfunction in frontal cortices (Deserno et al. 2020); this aspect of the DG inactivation effects reported here may be tentatively linked to frontal cortex function by considering that the hippocampal circuitry downstream of the DG (CA3, CA1 and subiculum) strongly projects to the medial PFC.

### 4.3 Neural Mechanisms Supporting the Contribution of the Dentate Gyrus to Place‐Outcome Learning

Our study suggests a role of the dDG in the learning of place‐reward associations. Specifically, targeting the dDG induced an impaired discrimination ability (Figure 2C) and reduced reward sensitivity (Figure 3E) during the initial formation or encoding of place‐outcome associations. The question remains, however, how the dDG contributes to the generation of place‐outcome representations.

This question may be approached, first, by considering results from DG studies using other learning paradigms. For instance, Kheirbek et al. (2013) found that optogenetic manipulation of the DG revealed that granule cells control exploratory drive and encoding of contextual fear memories, but not their retrieval. This is consistent with the effects we found on initial learning and alternation in an appetitive learning paradigm, as well as with a role for DG neurogenesis in improving spatial representations (Frechou et al. 2024). In a similar vein, optogenetic manipulation of somatostatin‐positive cells in the DG impaired encoding of spatial memories and conditioned place preference but, again, not their retrieval (Yen et al. 2022). One of the targets of our DREADD manipulation was the population of dDG mossy cells, which were recently shown to contribute to the encoding of space and the disambiguation of spatial context (Huang et al. 2024). The latter study differs from ours, however, in that different contexts were presented to head‐fixed mice in a virtual‐reality setup. Our study is more comparable to another study suggesting the DG to be acutely engaged during the discrimination of similar but distinct contexts when the animal experienced a negative outcome (van Dijk and Fenton 2018). This study agrees with our observations in that (i) the DG is engaged during spatial discrimination in a (ii) physically identical environment with relocated goal locations when (iii) animals are actively acquiring place‐outcome associations. In sum, our study is generally in line with these previous studies on the DG in that they are consistent with a role in acquisition and memory encoding, both in tasks where place or context becomes associated with positive or negative outcome.

Second, the potential mechanisms and pathways through which the dDG may be involved in place‐outcome learning must be considered. As the DG densely projects to area CA3 and, from there, indirectly affects area CA1 and the subiculum (reviewed in Amaral and Witter 1989), information about places and contexts associated with valuable outcomes may reach structures such as the ventral striatum and medial PFC, which are heavily implicated in reward expectancy, valuation of cues and actions, and goal‐directed behaviors (Alexander et al. 1986; Thierry et al. 2000; Voorn et al. 2004; Haber et al. 2006; Roberts et al. 2007). In line with this, Sasaki et al. (2018) found reward‐evoked activity in dentate cells at reward sites in rats performing a spatial working memory task, suggesting that DG cells encode place‐outcome representations. Importantly, reward‐evoked activity in the DG was associated with increased sharp‐wave‐ripple (SWR) rates in the CA3, where the lesioning of granule cells reduced SWR rates in the CA3. This study implicates that reward‐evoked network activity in the CA3 may be driven by the DG, where, in turn, recurrent collaterals from the CA3 may reinforce place‐outcome associations to support place‐reward learning in downstream areas. In line with this, CA1/subicular projections to the ventral striatum have been implicated in place‐reward learning (often called conditioned place preference; Ito et al. 2008; Lansink et al. 2009; Trouche et al. 2019). At the level of downstream area CA1, previous studies have shown that places associated with reward are encoded by place cells (Hollup et al. 2001; Hölscher et al. 2003; Lansink et al. 2012; Gauthier and Tank 2018; reviewed in Sosa and Giocomo 2021). For instance, the density and firing rate of place fields of CA1 cells are enhanced near goal locations associated with reward (Hollup et al. 2001; Hölscher et al. 2003; Gauthier and Tank 2018). Moreover, place fields in the CA1 are reduced in size at reward sites in a Y‐maze, especially when reward‐predicting cue lights were lit (Lansink et al. 2012), suggesting the encoding of places becomes more fine‐grained when they are associated with positive outcomes.

To what extent the dDG encodes purely spatial information or also transmits reward‐associated signals to these downstream areas remains to be investigated in more detail. Recent studies have suggested that dentate cells not only encode spatial information but also nonspatial information, including reward‐ (van Dijk and Fenton 2018; Sasaki et al. 2018), object‐ (GoodSmith et al. 2022) and sensory‐cue‐related information (Tuncdemir et al. 2022, Tuncdemir et al. 2023). For instance, GoodSmith et al. (2022) found that granule cells and mossy cells encoded a stable spatial map of the environment but also remapped in response to object‐related changes in the environment. This implicates that both DG subpopulations are capable of encoding spatial and non‐spatial information. In the current study, histology revealed dense DREADD+ expressing cells in the granule cell layer and hilar region (Figures 1F,G and S1A), the latter containing mostly mossy cells (Senzai and Buzsáki 2017; GoodSmith et al. 2017). Hilar mossy cells have been shown to strongly drive local DG interneurons (Jinde et al. 2012; Scharfman et al. 1990), thus affecting the granule cells that project onto the CA3 (reviewed in Amaral and Witter 1989), which in turn is thought to be important for associative learning of places with objects and sensory stimuli (Morris et al. 2012). The behavioral effects that we observed in the present study could therefore have arisen due to a disruption of a DG‐driven facilitation of the binding of motivationally salient events to exact places by downstream areas such as CA3, CA1‐subiculum (Lee and Jung 2017) and hence to ventral striatum and medial PFC. These hippocampal‐prefrontal‐striatal connections have been hypothesized to enable the prefrontal cortex to update behavioral task states and support flexible decision making (Brown et al. 2012, 2016; Brown and Stern 2014; Ferbinteanu 2016; Rusu and Pennartz 2020). This, in turn, suggests how the dDG dysfunction observed in our study may be associated with impaired decision‐making. Specifically, degraded spatial output from the hippocampus, relayed to the ventral striatum and prefrontal cortex, may have rendered the hippocampal‐prefrontal‐ventral striatal system unable to learn to choose the correct goal location, despite being associated with reward.

An alternative—but not mutually exclusive—mechanism holds that projections from the mesencephalon and/or brain stem to the DG contribute directly to reward‐dependent learning at the level of the DG itself (cf. Redondo et al. 2014). Results from Han et al. (2020) suggest that a glutamatergic projection from the ventral tegmental area to the DG contributes to opioid‐induced conditioned place preference, whereas Petter et al. (2023) presented evidence for a causal role of a dopaminergic projection from Locus Coeruleus to DG in operant reinforcement (self‐stimulation). Future studies will have to show which of these mechanisms may be involved in the type of place‐outcome learning within the same familiar environment as described here. Regardless of the precise mechanism, we note that a causal involvement of the DG in valence‐based spatial learning is functionally advantageous, because it is strategically positioned to influence a large, diverging cascade of brain structures receiving hippocampal output, including a wide range of medial temporal lobe structures and posterior cortices.

### 4.4 Conclusions

In conclusion, our study provides evidence for a mechanistic involvement of the dDG in learning place‐reward associations. Future studies will have to uncover the circuit‐level underpinnings of this role, in particular the interplay between the dDG and other brain regions.

## Author Contributions

J.L., A.S., and P.A. collected the behavioral data. J.L. analyzed the behavioral data with feedback from U.O. and C.M.A.P. J.L. and G.J.H.I.V. performed the surgeries. J.L., A.S., and P.A. performed histology. J.L. wrote the manuscript with extensive feedback from U.O. and C.M.A.P. C.A.O. designed the behavioral task. C.S.L., C.M.A.P., and C.A.O. created the general study design.

## Supporting information


**Figure S1.** Histological verification of DREADD mCherry+ expression in the dorsal DG (dDG). (A) Example animal 9 (A9) with bilateral DREADD mCherry expression in the dDG (left panel), DAPI expression (middle panel) and overlaid (right panel). Solid triangles indicate subregions of the hippocampus expressing DREADDs. (B) Close up of DREADD mCherry+ expression in the hilar region and granule cell layer of the left dDG. (C) Close up of DG axonal projections with DREADD mCherry+ expression in the left CA3, characterized by stripey structures. (D) Close up of DREADD mCherry+ expression in the left Subiculum and absent DREADD expression in the left CA1.

**Figure S2.** Histological verification of DREADD mCherry+ expression in the dorsal DG (dDG) for each individual rat. (A) Animal 1 with bilateral DREADD mCherry expression along the anterior–posterior axis of the dDG. The left panel represents the left hippocampus and the right panel represents the right hippocampus. The subsequent figure panels (B–K) show animals 2 to 11, respectively.

**Figure S3.** Quantification of the trials needed to reach the reversal criterion for each treatment and separation condition across learning phases. (A) Average number of reversals made for large and small sessions for animals with bilateral DREADD‐mCherry+ expression (*N* = 11) upon receiving treatment with saline (VEH, blue line) or CNO (orange line). (B) Average trials needed to reach the reversal criterion (TTC) per learning phase with baseline (VEH) treatment for Large (solid blackline) and Small (dotted black line) sessions. (C) Same as (B) but pooled across VEH and CNO treatment sessions. (D) TTC pooled across Large and Small sessions for each learning phase for VEH (blue line) and CNO treatment (orange line). (E) Same as panel B, but for Large sessions only. (F) Same as panel B, but for Small sessions only. (G) Average TTC (acquisition phase only) pooled across Large and Small sessions for each testing day number across animals (black solid line) and for individual rats (gray solid lines). Error bars indicate average task variables pooled across animals (mean ± SEM). Significance is indicated by *p < 0.05, **p < 0.01, ***p < 0.001. Shaded regions within panels indicate the panels already presented in Figure 2.

**Figure S4.** Number of trials to reach the reversal criterion (TTC) with saline (VEH, blue line) or CNO (orange line) for each learning phase for animals with bilateral DREADD+ expression in separate figure panels (A–K).

**Figure S5.** Behavioral task measures during location discrimination. (A) Proportion of trials where animals (*N* = 11) persisted in their choice relative to the previous trial for each learning phase for Large sessions only after saline (VEH, blue line) and CNO treatment (orange line). (B) Same as panel A, but for small sessions. (C) Proportion of incorrect trials of for each learning phase for large sessions. (D) Same as panel C, but for small sessions. (E) Response latency (in seconds, s) for each learning phase after VEH or CNO treatment for large sessions. (F) Same as panel E, but for small sessions. (G) Proportion of leftward choice trials for each learning phase after VEH or CNO treatment for Large sessions. (H) Same as panel G, but for Small sessions. Error bars indicate average task measures pooled across animals (mean ± SEM). Shaded blue regions for in the panels indicate panels shown in Figure 2.

**Figure S6.** Spatial distribution of nose poke responses and body speed of animals during task engagement with DG modulation. (A) Poke probability of bilateral DREADD+ animals (*N* = 11) to response locations 1 (L1) to 8 (L8) after saline (VEH, blue line) and CNO (orange line) treatment for Large sessions. The cued target and nontarget locations were alternated between L2 and L7 for subsequent reversals. Shaded error bars indicate average poke probability pooled across animals (mean ± SEM). (B) Same as panel (A), but for Small sessions. (C) Left: trajectory and body speed of a representative left trial for a Large VEH session of an animal (A9). Right: trajectory of a right trial for a Large CNO session of the same animal. Body speed s(in cm/s) is indicated with colors scaled by the color bar. (D) Body speed of a subset of bilateral DREADD+ animals (*N* = 8) for each learning phase for Large sessions after VEH and CNO treatment. All trials included. (E) Same as panel (C), but for a Small example session. (F) Same as (D), but for Small sessions. (G, I) Body speed of animals for correct trials per treatment condition for Large and Small sessions, respectively. (H, J) Body speed of animals for incorrect trials per treatment condition for Large and Small sessions, respectively. (K, M) Body speed of animals for leftward trials per treatment condition for Large and Small sessions, respectively. (L, N) Body speed of animals for rightward trials per treatment condition for Large and Small sessions, respectively. Error bars indicate average task variables pooled across animals (mean ± SEM). Significance is indicated by *p < 0.05, **p < 0.01. Shaded regions in panels (B) and (D) indicate plots already shown in Figure 2.

**Figure S7.** Modeling of choice behavior during discrimination of cued locations with large and small separation. (A) Representative example session of choice behavior per trial (black dots) and smoothened across 4 trials (back dotted line) of animal 8 (A8) during a Small separation session with saline treatment. The choice trace is overlaid with the modeled behavior from the Simple Reinforcement learning (RL) model (blue line) and the Perseverance RL (PRL) model (green line). The rewarded side is indicated with the red line (reward side switches after the animal made 9 out of 10 consecutive correct trials). (B) Median akaike information criterion (AIC) of the RL (blue square) and PRL model fits (green square) for Large and Small sessions. (C) Per‐session correlations (R) between the observed and modeled choices per condition as fitted by the RL (blue line) and PRL model (green line). (D) Average learning rate (alpha) for each learning phase with Large (solid line) or Small (dotted line) sessions. (E) Same as panel D, but for the average reward sensitivity (beta). (F) Same as panel D, but for the average perseverance rate (delta). Error bars indicate average task variables pooled across animals (mean ± SEM). Significance is indicated by **p < 0.01, ***p < 0.001.

**Table S1.** Statistical tests. Statistical comparisons were not carried out if the number of animals was below 2.

## Data Availability

The data that support the findings of this study are available from the corresponding author upon reasonable request.
