# Supplementary figures and images for: Transient DREADD Manipulation of the Dorsal Dentate Gyrus in Rats Impairs Initial Learning of Place‐Outcome Associations
Source: Hippocampus. 2025 May 6;35(3):e70014. doi: 10.1002/hipo.70014 (PMC12053149; doi:10.1002/hipo.70014)

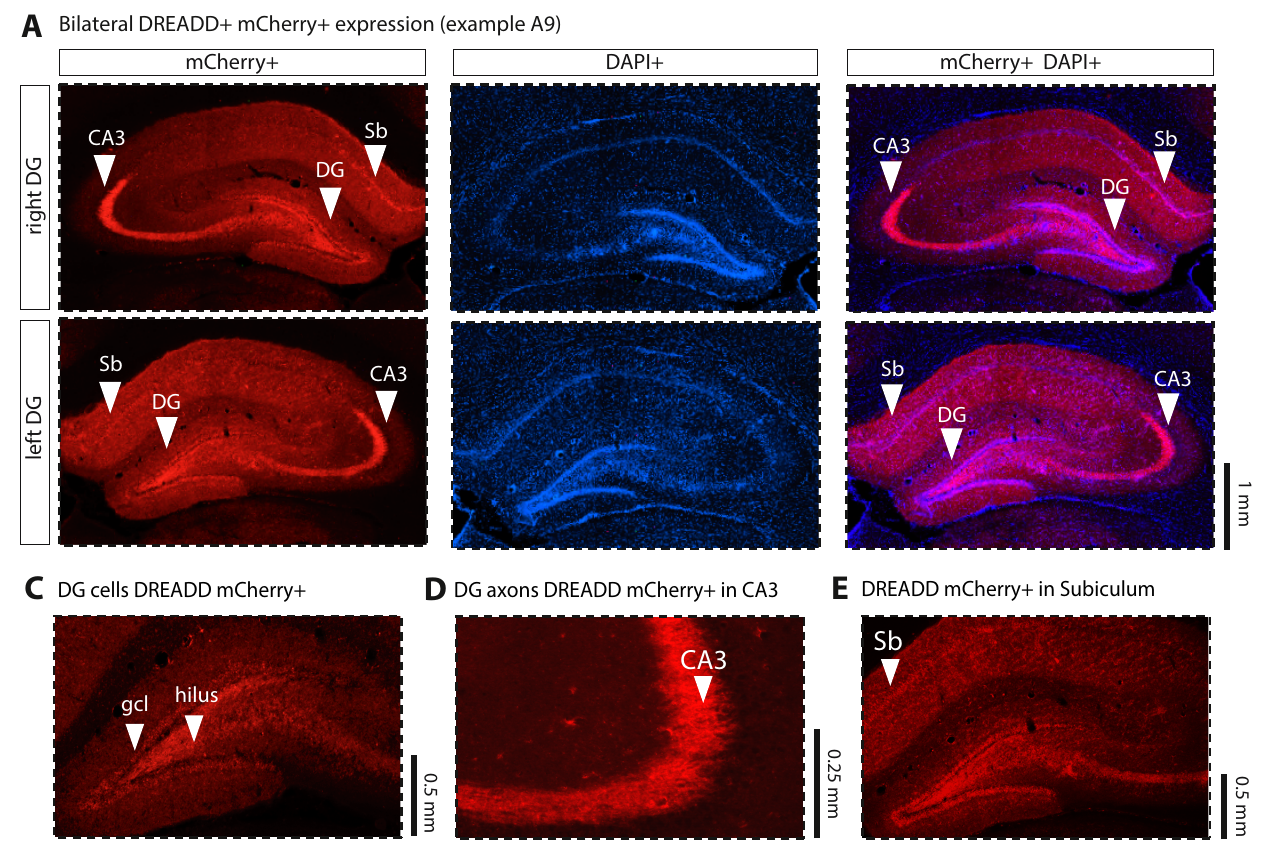

Supplement: Supplementary file 1 — Figure S1. Histological verification of DREADD mCherry+ expression in the dorsal DG (dDG). (A) Example animal 9 (A9) with bilateral DREADD mCherry expression in the dDG (left panel), DAPI expression (middle panel) and overlaid (right panel). Solid triangles indicate subregions of the hippocampus expressing DREADDs. (B) Close up of DREADD mCherry+ expression in the hilar region and granule cell layer of the left dDG. (C) Close up of DG axonal projections with DREADD mCherry+ expression in the left CA3, characterized by stripey structures. (D) Close up of DREADD mCherry+ expression in the left Subiculum and absent DREADD expression in the left CA1. [file HIPO-35-0-s003.tiff]

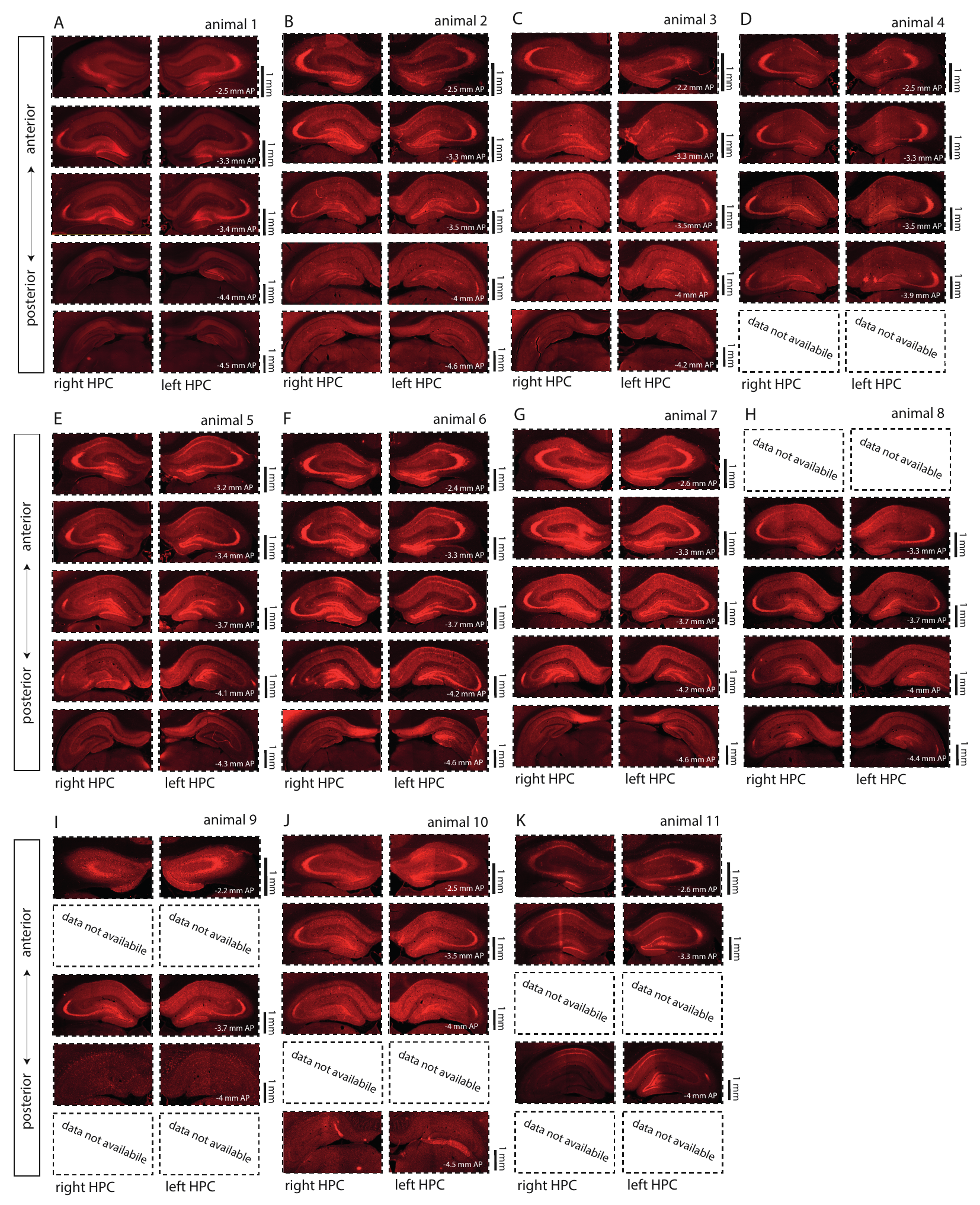

Supplement: Supplementary file 2 — Figure S2. Histological verification of DREADD mCherry+ expression in the dorsal DG (dDG) for each individual rat. (A) Animal 1 with bilateral DREADD mCherry expression along the anterior–posterior axis of the dDG. The left panel represents the left hippocampus and the right panel represents the right hippocampus. The subsequent figure panels (B–K) show animals 2 to 11, respectively. [file HIPO-35-0-s008.tiff]

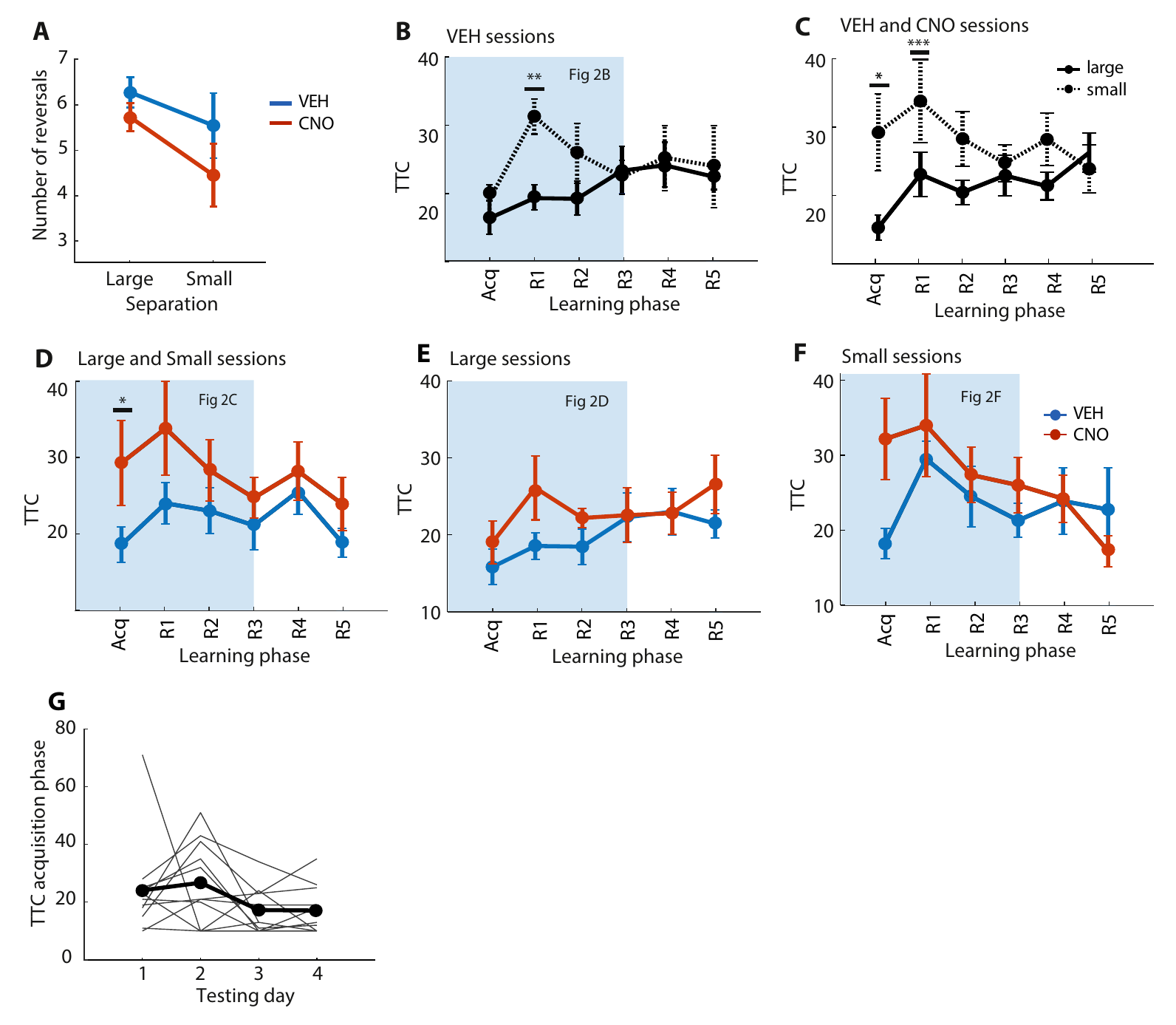

Supplement: Supplementary file 3 — Figure S3. Quantification of the trials needed to reach the reversal criterion for each treatment and separation condition across learning phases. (A) Average number of reversals made for large and small sessions for animals with bilateral DREADD‐mCherry+ expression (N = 11) upon receiving treatment with saline (VEH, blue line) or CNO (orange line). (B) Average trials needed to reach the reversal criterion (TTC) per learning phase with baseline (VEH) treatment for Large (solid blackline) and Small (dotted black line) sessions. (C) Same as (B) but pooled across VEH and CNO treatment sessions. (D) TTC pooled across Large and Small sessions for each learning phase for VEH (blue line) and CNO treatment (orange line). (E) Same as panel B, but for Large sessions only. (F) Same as panel B, but for Small sessions only. (G) Average TTC (acquisition phase only) pooled across Large and Small sessions for each testing day number across animals (black solid line) and for individual rats (gray solid lines). Error bars indicate average task variables pooled across animals (mean ± SEM). Significance is indicated by *p < 0.05, **p < 0.01, ***p < 0.001. Shaded regions within panels indicate the panels already presented in Figure 2. [file HIPO-35-0-s001.tiff]

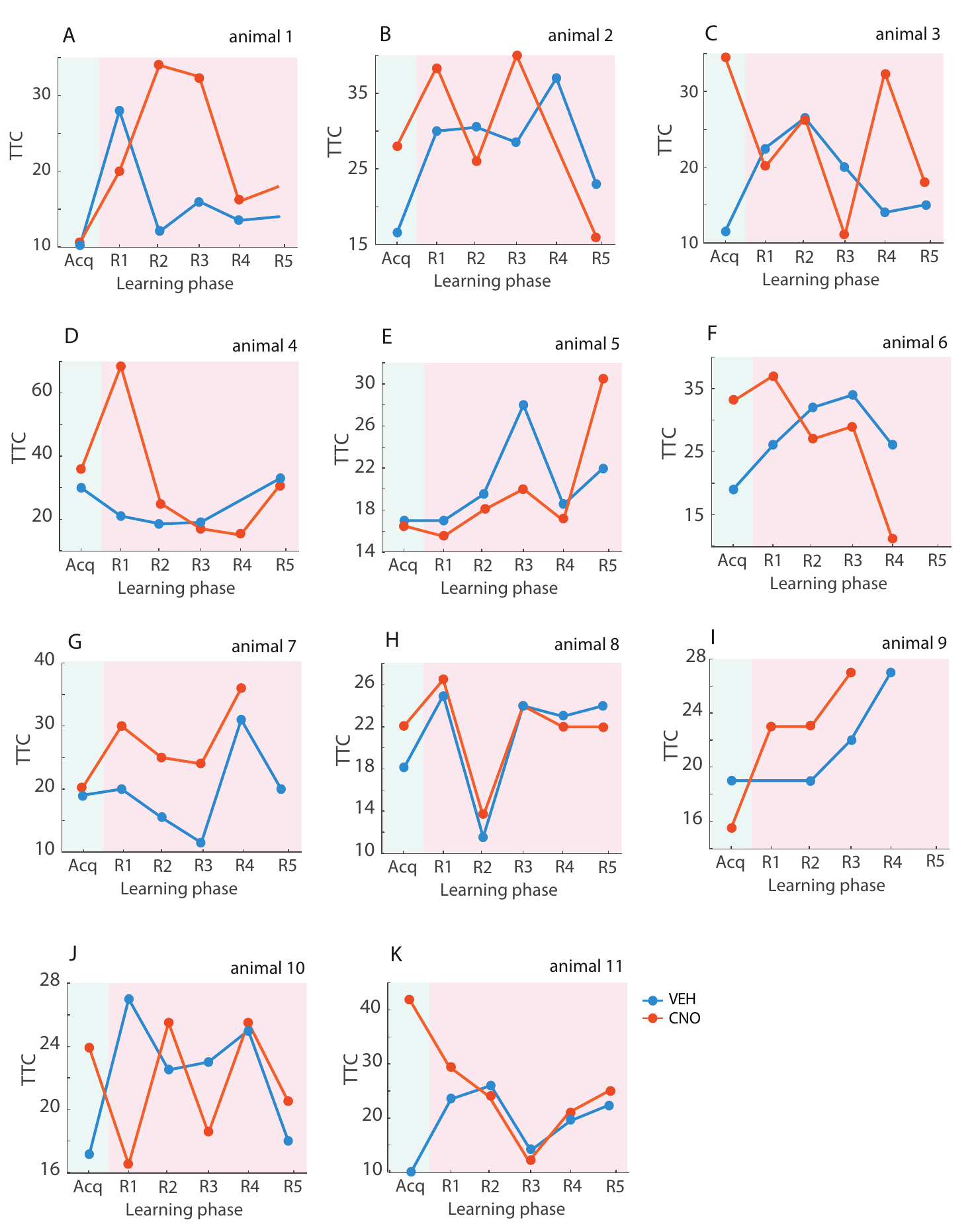

Supplement: Supplementary file 4 — Figure S4. Number of trials to reach the reversal criterion (TTC) with saline (VEH, blue line) or CNO (orange line) for each learning phase for animals with bilateral DREADD+ expression in separate figure panels (A–K). [file HIPO-35-0-s005.tiff]

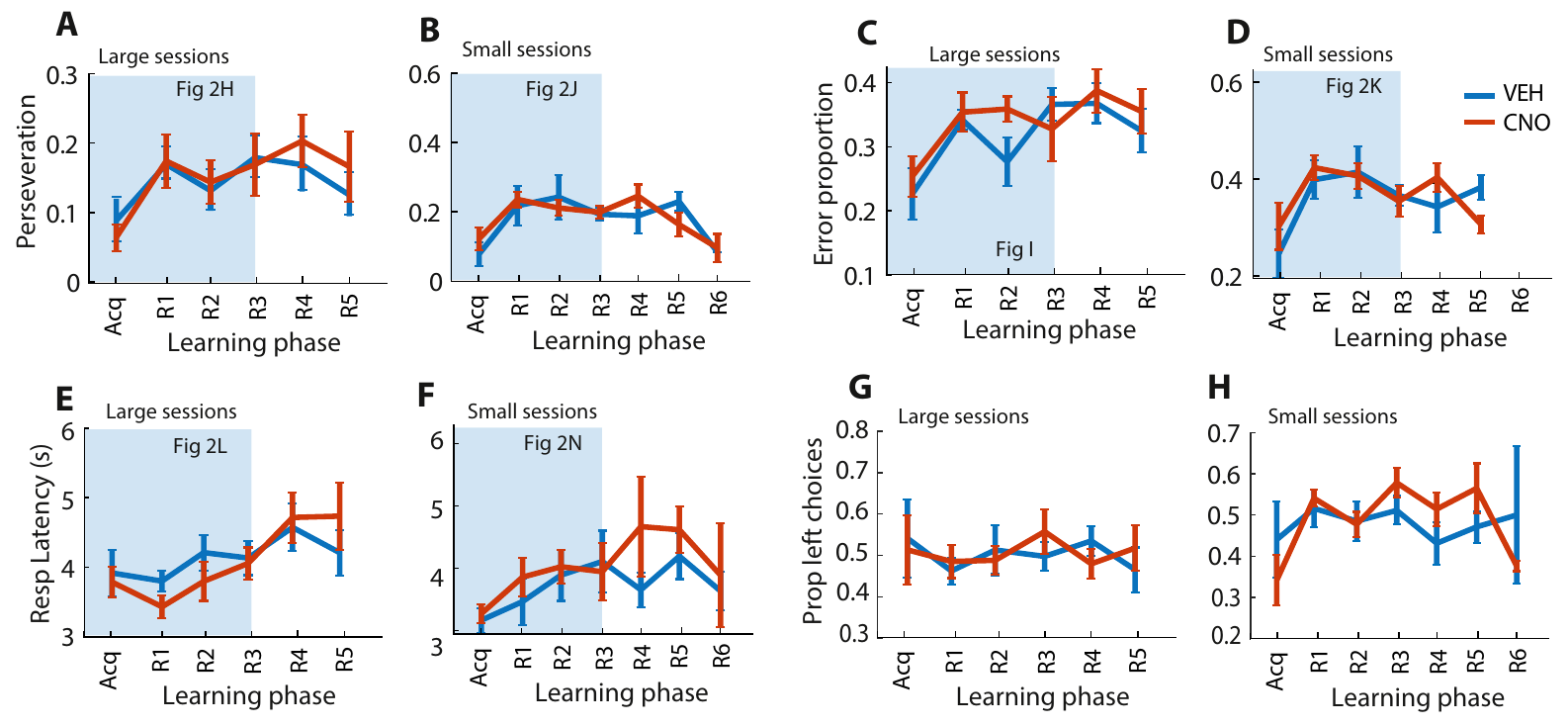

Supplement: Supplementary file 5 — Figure S5. Behavioral task measures during location discrimination. (A) Proportion of trials where animals (N = 11) persisted in their choice relative to the previous trial for each learning phase for Large sessions only after saline (VEH, blue line) and CNO treatment (orange line). (B) Same as panel A, but for small sessions. (C) Proportion of incorrect trials of for each learning phase for large sessions. (D) Same as panel C, but for small sessions. (E) Response latency (in seconds, s) for each learning phase after VEH or CNO treatment for large sessions. (F) Same as panel E, but for small sessions. (G) Proportion of leftward choice trials for each learning phase after VEH or CNO treatment for Large sessions. (H) Same as panel G, but for Small sessions. Error bars indicate average task measures pooled across animals (mean ± SEM). Shaded blue regions for in the panels indicate panels shown in Figure 2. [file HIPO-35-0-s006.tiff]

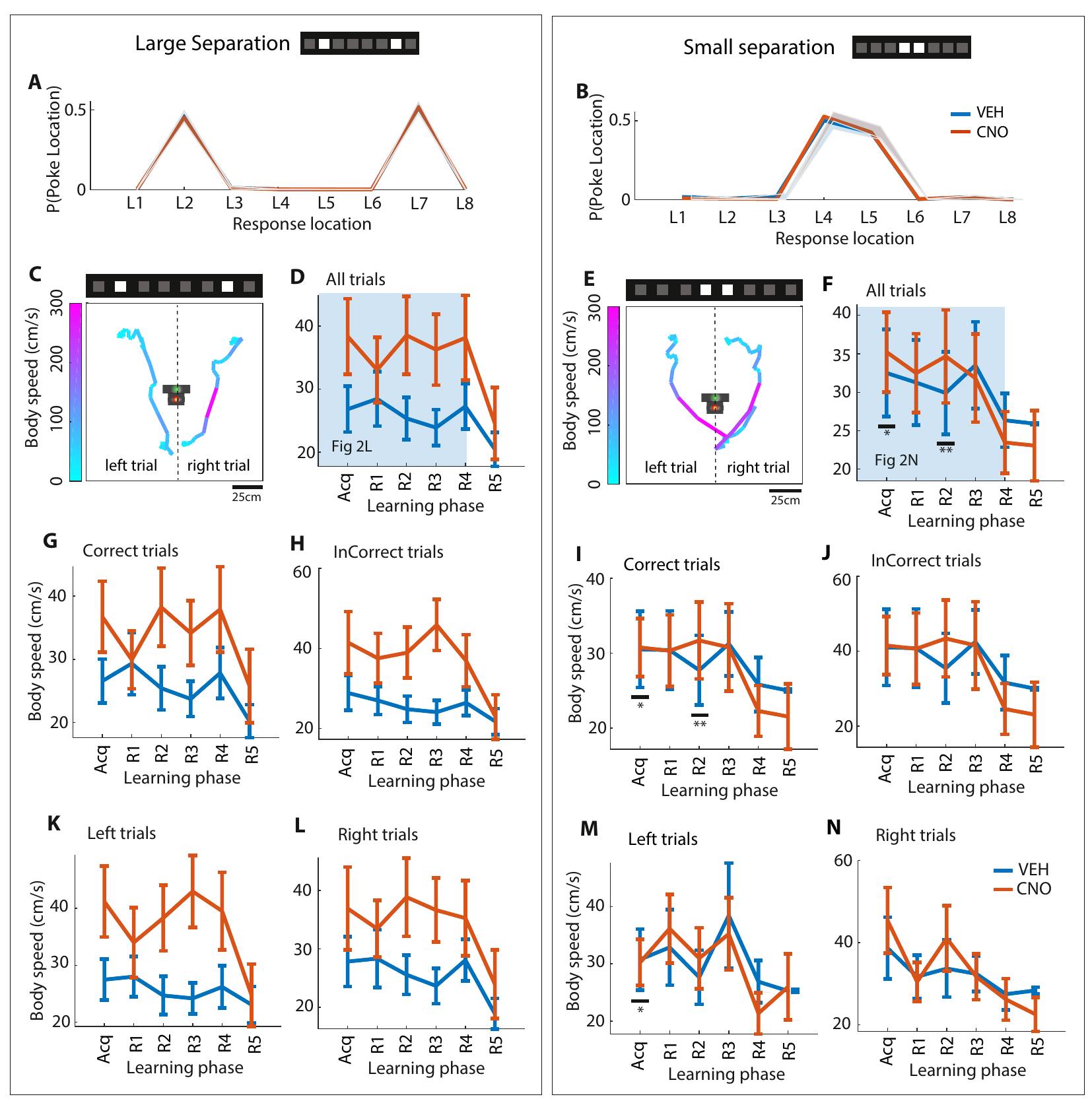

Supplement: Supplementary file 6 — Figure S6. Spatial distribution of nose poke responses and body speed of animals during task engagement with DG modulation. (A) Poke probability of bilateral DREADD+ animals (N = 11) to response locations 1 (L1) to 8 (L8) after saline (VEH, blue line) and CNO (orange line) treatment for Large sessions. The cued target and nontarget locations were alternated between L2 and L7 for subsequent reversals. Shaded error bars indicate average poke probability pooled across animals (mean ± SEM). (B) Same as panel (A), but for Small sessions. (C) Left: trajectory and body speed of a representative left trial for a Large VEH session of an animal (A9). Right: trajectory of a right trial for a Large CNO session of the same animal. Body speed s(in cm/s) is indicated with colors scaled by the color bar. (D) Body speed of a subset of bilateral DREADD+ animals (N = 8) for each learning phase for Large sessions after VEH and CNO treatment. All trials included. (E) Same as panel (C), but for a Small example session. (F) Same as (D), but for Small sessions. (G, I) Body speed of animals for correct trials per treatment condition for Large and Small sessions, respectively. (H, J) Body speed of animals for incorrect trials per treatment condition for Large and Small sessions, respectively. (K, M) Body speed of animals for leftward trials per treatment condition for Large and Small sessions, respectively. (L, N) Body speed of animals for rightward trials per treatment condition for Large and Small sessions, respectively. Error bars indicate average task variables pooled across animals (mean ± SEM). Significance is indicated by *p < 0.05, **p < 0.01. Shaded regions in panels (B) and (D) indicate plots already shown in Figure 2. [file HIPO-35-0-s002.tiff]

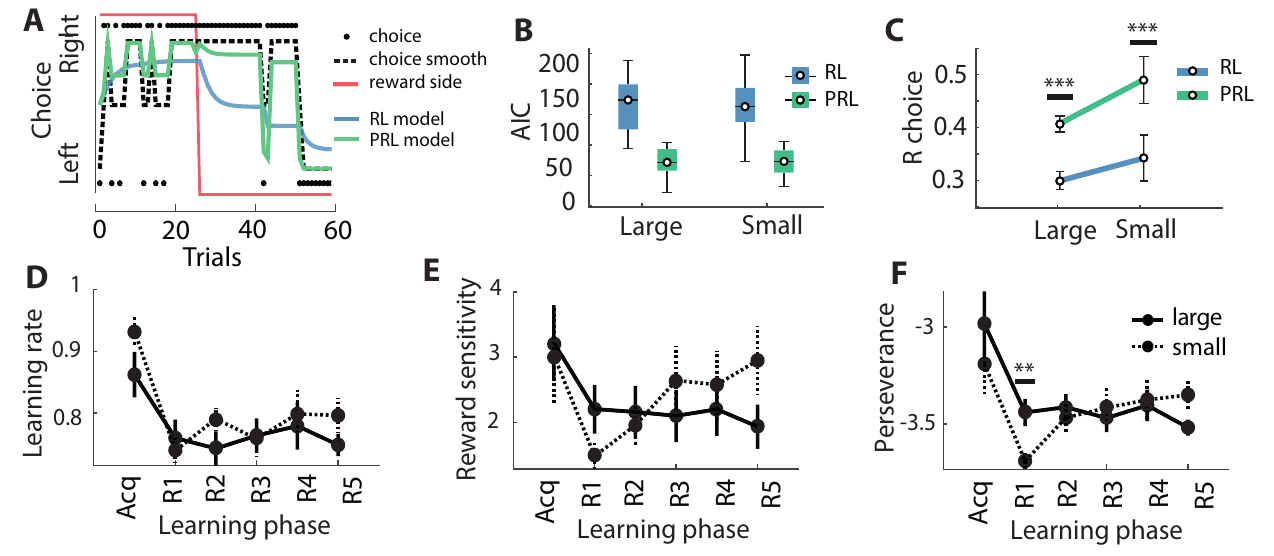

Supplement: Supplementary file 7 — Figure S7. Modeling of choice behavior during discrimination of cued locations with large and small separation. (A) Representative example session of choice behavior per trial (black dots) and smoothened across 4 trials (back dotted line) of animal 8 (A8) during a Small separation session with saline treatment. The choice trace is overlaid with the modeled behavior from the Simple Reinforcement learning (RL) model (blue line) and the Perseverance RL (PRL) model (green line). The rewarded side is indicated with the red line (reward side switches after the animal made 9 out of 10 consecutive correct trials). (B) Median akaike information criterion (AIC) of the RL (blue square) and PRL model fits (green square) for Large and Small sessions. (C) Per‐session correlations (R) between the observed and modeled choices per condition as fitted by the RL (blue line) and PRL model (green line). (D) Average learning rate (alpha) for each learning phase with Large (solid line) or Small (dotted line) sessions. (E) Same as panel D, but for the average reward sensitivity (beta). (F) Same as panel D, but for the average perseverance rate (delta). Error bars indicate average task variables pooled across animals (mean ± SEM). Significance is indicated by **p < 0.01, ***p < 0.001. [file HIPO-35-0-s004.tiff]
